# Spatial transcriptomics on an expanded dataset at the brain-electrode interface: exploration of variability and identification of novel biomarkers

**DOI:** 10.3389/fnins.2026.1852774

**Published:** 2026-06-15

**Authors:** Bhavna Gupta, Anirban Chakraborty, Akash Saxena, Michael G. Moore, Quentin A. Whitsitt, Erin K. Purcell

**Affiliations:** 1 Department of Biomedical Engineering, Michigan State University, East Lansing, MI, United States; 2 Institute for Quantitative Health Science and Engineering, Michigan State University, East Lansing, MI, United States; 3 Department of Electrical and Computer Engineering, Michigan State University, East Lansing, MI, United States

**Keywords:** biomarkers, chronic implant, differential gene expression, foreign body reaction, neural device, silicon microelectrode array, spatial transcriptomics

## Abstract

The foreign body reaction to implanted electrodes in the brain has long been recognized as a major challenge impacting the performance and reliability of indwelling neurotechnologies. Spatially resolved transcriptomic approaches have enabled high-resolution mapping of cellular and molecular dynamics at the device-tissue interface, yielding novel insight into both acute and chronic tissue responses. Recent whole-transcriptome profiling methods generate exceptionally dense gene expression datasets from individual samples, offering unprecedented resolution and analytical power. Yet, limited studies have explored aggregated results from larger datasets and sample-to-sample variation within an implanted cohort using such techniques due to high costs and complicated downstream analyses. In this work, we provide a comprehensive report of spatial transcriptomics data collected from an expanded cohort of rats (*n* = 14 rats) implanted with silicon microelectrode arrays in the motor cortices for 1 week (acute) and 6 weeks (chronic). This larger dataset enabled us to explore the variation in results across samples, assess outliers, and examine potential batch effects. We employed differential expression analysis to identify top differentially expressed genes (DEGs) in spatially defined regions at the device-tissue interface to reveal novel biomarkers in the aggregated dataset. We assessed sample-to-sample variabilities, and applied a factorization strategy to identify prominent cell-type contributors of the top DEGs. Using network-based co-expression analysis, we identified gene modules, hub genes, and central regulatory processes governing the device-tissue interface. Our results show: (a) greater variation of top DEGs across samples at the 1-week time point with notable microglial and astroglial cell-type contributors, (b) lower variation of top DEGs across samples and a shift to prominent astroglial cell-type contributors at the 6-week time point, and (c) novel biomarkers that suggest major macrophage- and microglial mediated processes and homeostasis events at the 1-week time point, and greater tissue remodeling, apoptotic and synaptic changes at the 6-week time point. These findings support previous ideas on the evolving tissue response to implanted devices, and present novel details on biomarkers, biological processes and sample variation. Additionally, this study provides a framework for assessing larger datasets employing high-dimensional spatial transcriptomics and highlight key considerations related to across-sample variability and batch effects.

## 1 Introduction

Implantable electrodes in the brain have revolutionized the study of neural function, and treatment of neurological conditions (BrainGate Trials, 2025; Collinger et al., 2013; Evangelisti et al., 2023; Nijhuis et al., 2023; Quinn et al., 2015; Willsey et al., 2025; Yin et al., 2023). Modern assistive technologies, including brain computer interfaces (BCIs) and neuromodulation systems, often rely on accurate neural signal detection to perform effectively. Yet, electrical recordings from implanted neural probes often reveal a trend in signal decline over subsequent weeks and months (Chestek et al., 2011; Kozai et al., 2015; McCreery et al., 2002; Perge et al., 2013). Such variabilities can be compounded by additional undesirable effects, such as insertional trauma, shifting stimulation thresholds, and off-target consequences. Detailed investigations of the device-tissue interface are warranted to better understand the chronic foreign-body tissue response to implanted electrode arrays, driving the use of newer and more sophisticated research techniques (Cabrera et al., 2019; Grill, 2008; Gupta et al., 2024; Salatino et al., 2017a; Thompson et al., 2020). Neuronal density and astrocytic expression of glial fibrillary acidic protein (GFAP) are typically assessed to characterize the biological response and tissue health at the device-tissue interface (Biran et al., 2005). However, traditional histology is relatively low throughput due to the pre-selection of a small number of biomarkers to provide minimal information on the biological mechanisms governing the tissue response (Michelson et al., 2018). Recent use of high throughput RNA-sequencing and spatial transcriptomics techniques to map transcriptional changes in cells surrounding devices (Marx, 2021) have revealed new information about the foreign body reaction (FBR) to implanted electrodes and signal quality (Thompson et al., 2021; Thompson et al., 2023; Whitsitt et al., 2021; Whitsitt et al., 2022).

Previous work by Thompson et al. using laser capture microscopy extracted interfacial (≤100 μm from device site) and distal tissue (∼500 μm from device site) samples from silicon microelectrodes for RNA-sequencing. Hundreds of differentially expressed genes (DEGs) were revealed when comparing tissue close to implanted electrodes relative to non-implanted naïve tissues (Thompson et al., 2021). Specific DEGs were significantly upregulated or downregulated across timepoints (24 h, 1 week, and 6 weeks) and classified based on known molecular and cellular mechanisms: reactive microglia/inflammation (e.g., *Gpnmb, Cx3cr1, Tnfrsf1a, C3*), oligodendrocyte metabolism and myelin maintenance (e.g., *Olig2*, *Plp1*, *Tf, Mbp*, *Cnp, Fth1*), neuronal function and plasticity (e.g., *Nefh, Camk2a*, *Snap25, Arc*), astrocyte activation and fibrosis (e.g., *Gfap, Aqp4, Vim, Ptbp1 Best1*), lysosomal activity (*Ctss, Ctsb*), proliferation (*Csf1r*), and phagocytosis (*Dock8*). These RNA-seq results corroborate other similar findings (Ereifej et al., 2018; Golabchi et al., 2018; Prasad et al., 2014; Wellman et al., 2019) on cellular and molecular changes (as gene expression differences) in the biological environment surrounding implanted electrodes in the brain. For example, highly expressed inflammatory and phagocytic genes via reactive microglia continued over 6-weeks post-implantation, emphasizing the presence of neurotoxic mechanisms. Steady upregulation in oligodendrocyte genes associated with iron metabolism, cellular identity and myelination (Chen et al., 2021; Chen et al., 2023; Wellman et al., 2020) possibly indicates an added demand for metabolically taxing remyelination and oligodendrocyte turnover at the device interface over time. Decreased regulation of neuronal genes related to synaptic function and dendritic spine maintenance supports the idea that neuronal damage/dysfunction-associated mechanisms contribute to reduced signal quality (Gregory et al., 2023).

Several groups have advanced our understanding of the biological response to implanted electrodes and its temporal dynamics through genetic and proteomic analyses techniques (Druschel et al., 2024; Falcone et al., 2018; Hernandez-Reynoso et al., 2023; Joseph et al., 2021; Pflüger et al., 2020; Song et al., 2024a; Song et al., 2024b; Thompson et al., 2026; Zhao et al., 2025). Early studies characterizing differential gene expression during the acute (24-h post implantation) (Bedell et al., 2020) and chronic (4-, 8-, and 16-weeks post implantation) (Song et al., 2023) phases of the immune response to intracortical microelectrodes primarily relied on targeted bulk gene expression profiling. More recent works have sought to assess and/or mitigate the neuroinflammatory response by examining gene expression at the interface in a variety of conditions, including genetically modified models (Song et al., 2022), implantation-accompanied injuries (e.g., stab wounds and craniotomies) (Huff et al., 2026), and electrode-design modifications (such as size, coatings, and materials) (Duncan et al., 2024; Kim et al., 2023; Pflüger et al., 2020; Thompson et al., 2026). Additionally, emerging spatially resolved transcriptomics methods have enabled whole-tissue section mapping of gene expression levels, providing an advantageous framework for assessing spatial and temporal heterogeneity of tissue responses to implanted electrodes (Whitsitt et al., 2021; Whitsitt et al., 2024). Whitsitt et al. compared DEGs between implanted and non-implanted tissue at 24-h, 1-week, and 6-weeks to reveal distinct temporal changes in both the spatial extent of gene expression and the evolving tissue response, motivating the need for deeper investigations of acute-to-chronic FBR progression using novel whole-tissue mapping of gene expression.

A key limitation of transcriptomics assays is their typically high cost: in turn, many studies report relatively limited sample sizes (Whitsitt et al., 2021; Whitsitt et al., 2024), which leaves open questions regarding the repeatability and consistency of results and underlying sources of variation (both technical and biological). In this work, we analyze our previously reported spatial transcriptomics (Whitsitt et al., 2024) with an additional cohort of newly collected tissue samples from rats implanted with silicon microelectrode arrays in the motor cortex for 1 week (*n* = 7 rats) or 6 weeks (*n* = 7 rats). This allowed us to explore the variation in results across samples, investigate outliers, and consider batch effects in a larger data set. We identified biomarkers associated with the device-tissue interface in the larger data set by employing differential expression analysis to uncover top DEGs in spatially defined regions across aggregated samples for each time point. We investigated sample-to-sample variations and used a factorization strategy to identify the prominent cellular type contributors to top DEGs. Additionally, we applied network-based differential co-expression analysis to identify gene modules and central biological processes that govern the device-tissue interface. The results herein show that: (a) at 1 week post-implantation, top DEGs have greater variation across samples with prominent microglial and astroglial cellular contributors; meanwhile, sample-to-sample variations in top DEGs decrease, with greater astroglial contributions, at the 6-week time point; and, (b) specific biomarkers at each time point imply that the acute FBR experiences strong marcophage- and microglial-mediated biological interactions and homeostasis events, while the chronic phase encounters greater tissue remodeling, apoptotic and synaptic changes. These results corroborate previous ideas on tissue response progression from acute to chronic in the implanted brains, while presenting novel biomarkers of neural injury, inflammation, and implant-driven immune response. We provide analysis and discussion on the variation of specific biomarkers between samples, and we consider approaches to identify and address potential batch effects. The findings here further the understanding of the cellular and molecular changes taking place in implanted brains.

## 2 Materials and methods

### 2.1 Surgical implantation, brain extraction, and tissue sectioning

Previously published methods were followed for surgical implantation of devices and brain extraction (Thompson et al., 2021; Whitsitt et al., 2021). Briefly, single-shank, planar silicon Michigan-style microelectrode arrays (A1 × 16–3 mm-100–703-CMLP, 15 μm thickness, NeuroNexus Inc., Ann Arbor, MI) were implanted in the motor cortices of adult male Sprague Dawley rats (∼12 weeks old). Rats were anesthetized under isoflurane (∼2.0% in oxygen) and devices were stereotaxically lowered in the M1 region of motor cortices (+3.0 mm AP, +2.5 mm ML from Bregma, and 2.0 mm deep from cortex). A head cap of dental acrylic was made to close the surgical site and secure the device in place. Meloxicam (2 mg/kg, subcutaneously) and bupivacaine (topically) were administered as post-operative analgesics. Devices were implanted in *n* = 14 rats (*n* = 7 rats for 1-week, and *n* = 7 rats for 6-weeks). Two experimenters were involved in collecting tissue (*n* = 14 rat brains) reported here: tissue sections from *n* = 4 rats were collected by experimenter 1 and have been reported previously (Whitsitt et al., 2024), meanwhile tissue from *n* = 10 rats were newly collected by experimenter 2. At the terminal timepoint, rats were euthanized by an overdose intraperitoneal delivery of sodium pentobarbital. For previously collected tissue sections (*n* = 4 rats), brains were rapidly extracted after decapitation post-euthanasia and cryo-embedded immediately in a chamber containing liquid nitrogen. For newer collected tissue (*n* = 10 rats) cardiac perfusions with 100 mL of sterile Dulbecco’s Phosphate Buffered Saline (Sigma-Aldrich, D8537–100ML) were carried out post-euthanasia, and brains were rapidly extracted after decapitation. Brains were cryo-embedded immediately after removal in a chamber containing dry ice. For all samples, brain tissue was cryosectioned at a depth ∼1,000 μm from the cortical surface. One tissue section of the implanted hemisphere from each animal was mounted in the designated capture area of the Visium Spatial Gene Expression slide (10x Genomics, Pleasanton CA). Each tissue section was trimmed prior to mounting to fit within the fiducial frame boundaries of the Visium capture areas. All animal procedures were approved by the Michigan State University Animal Care and Use Committee.

### 2.2 Immunohistochemistry (IHC), spatial transcriptomics (ST), and RNA sequencing

The spatial transcriptomics assay, along with IHC, was followed based on the vendor protocol (10x Genomics), and as reported previously (Whitsitt et al., 2021; Whitsitt et al., 2024). Briefly, the spatial gene expression platform by 10x Genomics offers a Visium Spatial Gene Expression slide containing four capture areas (6.5 × 6.5 mm) marked by fluorescent fiducial frames. Each capture area further consists of ∼5,000 spatially barcoded oligonucleotides, organized as spots (each with a 55 μm diameter) placed 100 μm apart (center-to-center), to capture mRNA from mounted tissue.

In the workflow, immunohistochemistry and imaging are performed prior to sequencing in order to overlay the ST data on immunostained wide-field images. Tissue sections placed on the Visium Spatial Gene Expression slide were fixed in chilled methanol (at −20° Celsius) and blocked with Bovine Serum Albumin (BSA). Tissue was immunostained for GFAP primary antibody (Mouse Monoclonal GFAP antibody, 1:400, Millipore Sigma, St. Loius, Mo, Cat. #: A11034) and neuronal nuclei (NeuN) primary antibody (rabbit Polyclonal NeuN antibody, 1:100, Abcam, Cambridge, MA, Cat. #: 104225). Secondary antibodies of AlexaFluor 647 (Anti-mouse IgG, Invitrogen, Eugene, OR Cat. #: A21235) for conjugation to GFAP primary, and AlexaFluor 488 (Anti-rabbit IgG, Invitrogen, Eugene, OR, Cat. #: A11034) for conjugation for NeuN primary were used. A counterstain of Hoechst 33342 (1:10,000, Life Technologies Corp, Eugene, OR, Cat. #: H3570) was employed to label all nuclei. After staining, the Visium slide was coverslipped, and tissue imaging was conducted at the Michigan State University Center for Advanced Microscopy. A Leica Stellaris 5 CLSM confocal microscope with a motorized headstage was employed to image individual tiles (10x magnification) of each capture area containing tissue. A final wide-field image was generated by reconstructing, or stitching, the individual image tiles via the automated Leica software.

After imaging, the coverslip on the Visium Spatial Gene Expression slide was removed, and subsequent steps for tissue permeabilization and complementary DNA (cDNA) synthesis were performed. Briefly, tissue sections on the Visium slide were enzymatically permeabilized to release and capture the polyadenylated mRNA of cells overlying the spots (containing barcoded oligonucleotides) in the capture areas. The optimal permeabilization time was determined in a prior experiment as 18 min (Whitsitt et al., 2021). Next, reagents for reverse transcription were added to the slide to extend the capture oligonucleotides based on the bound mRNA sequence. A series of template switching and second strand synthesis then produced a spatially barcoded, full-length cDNA from the captured mRNA. This was followed by denaturation and cDNA transfer from each capture area into a corresponding DNA/RNA LoBind microcentrifuge tube, producing 4 sample tubes for each capture area containing tissue. A small amount (∼1 μL) of the cDNA from each sample was transferred to a quantitative polymerase chain reaction (qPCR) plate for amplification. Based on the qPCR amplification plot, a quantification cycle (Cq) value was recorded for each sample at ∼25% of the peak fluorescence value. The Cq value determined the number of cycles needed for further cDNA amplification of the samples, ensuring adequate mass for library construction. After amplification, the cDNA was cleaned up using SPRIselect (Beckman Coulter Inc., Brea, CA), and samples were transferred to new microcentrifuge tubes for library construction and sequencing.

Samples containing cleaned cDNA were delivered to the University of Michigan Advanced Genomics core for library preparation and sequencing. Assessment of cDNA quality was carried out using the Tapestation 2200 (Agilent) device. Based on the vendor protocol (10x Genomics), cDNA was prepared into a library for subsequent sequencing. The LabChip GX (PerkinElmer) was used for assessment of the prepared libraries. The final libraries were pooled and paired-end sequencing was performed using the Illumina NovaSeq 6000 system. Raw sequencing data were converted into de-multiplexed Fastq files with the Bcl2fastq2 conversion software. Lastly, the SpaceRanger pipeline (10x Genomics) was employed to align the sequencing reads to a reference genome and produce count matrices that were used to quantify the number of reads related to each gene. All samples were run with the reference genome, mRatBN7-2-2024-A, before aggregation to ensure stability of Ensembl IDs across samples. The AGGR function in SpaceRanger (10x Genomics) was employed to aggregate the dataset presented in this work.

### 2.3 Data analysis and computational methods

#### 2.3.1 Differential expression analysis of sequenced genes

The sequenced data were made available in .cloupe files, accessible by the 10x Genomics software “Loupe Browser,” and raw .fastq files. The .cloupe files can combine spatial-barcodes in the sequencing data with the wide-field immunostained image (.tif) of a capture area for a visual representation of gene expression on a stained tissue section. This is facilitated by the fiducial frame (visible on the red channel, 594 nm, while imaging) that defines a capture area, matching the sequencing reads to their corresponding location on the Visium slide. Once a .cloupe file is loaded in Loupe Browser, the gene expression (overlaid on IHC images) can be analyzed by selecting clusters of spots manually. The gene expression levels between clusters can be compared to generate lists of differentially expressed genes (DEGs) demonstrating upregulation and/or downregulation of detected genes, i.e., differential expression analysis. These DEG lists generated by LoupeBrowser contain values with the Log_2_Fold Change (LFC), and a *p*-value, adjusted using the Benjamini-Hochberg correction for multiple values, for each gene. The software also uses a correction factor for differences in number of spots selected in comparisons (LoupeBrowser, 2025). The LFC is a ratio of normalized mean gene Unique Molecular Identifier (UMI) counts in a cluster relative to other selected clusters. It is important to note that RNA sequencing identifies counts of gene transcripts in each spot of the capture area in a Visium slide, and these reads (tagged with a spatial barcode) also have a UMI that identifies individual molecules of RNA, ensuring that each count can be matched to its original molecule. A single count contributing to the differential expression analysis must have a UMI, spatial barcode and gene annotation (recognizing which gene that RNA molecule belongs to). Differential expression analysis of genes and representative spatial gene expression images reported in this work were generated by loading an aggregated dataset in the Loupe Browser software by 10x Genomics.

While subtle deviations in technical details (such as operator/user dependent variability, individuality of Visium slides, processing dates, and tissue conditions), the available options for correction were either inconsistent with our downstream analysis needs or failed to produce an observable correction (discussed further below). We employed the standard SpaceRanger aggregation produced data, which is suitable for downstream analysis of differential expression and co-expression. We proceeded by processing all 14 separate Visium datasets’ raw sequencing data using the SpaceRanger count pipeline (v4.0.14, 10x Genomics, Pleasanton, CA). This pipeline matched raw FASTQ reads with a single, consistent rat reference transcriptome (refdata-gex-mRatBN7-2-2024-A) to generate a feature-spot barcode matrix for each sample. DEGs with LFC ≥ 0.6 or ≤− 0.6, and adjusted *p* < 0.05 were considered significant. The p-value was transformed to -log_10_(p-value), following standard convention, for better visualization of the data. The volcano plot x-axis was plotted to contain the LFC, and y-axis was plotted with adjusted *p*-values. Significance thresholds are indicated by dotted lines, and significant DEGs are highlighted in red in the volcano plots. Volcano plots to visualize DEGs were created using GraphPad Prism 11 for Windows (GraphPad Software, Boston, MA, United States).

Manual selections of the spatially mapped regions of the ST tissue sections are possible via the analysis software LoupeBroswer (10x Genomics), enabling investigations of groups of DEGs at selected locations within and between tissue samples. First, for each sample, the general location of the implant was identified by qualitative observations of GFAP expression patterns in IHC images and the overlaid *Gfap* spatial gene expression distribution obtained from spatial transcriptomics. Second, the center point of implant injury was selected by qualitatively observing: (a) maximum GFAP intensity and/or visible tissue scarring of the device tract, and (b) *Gfap* heatmaps of overlaid spatial transcriptomics data. Using the spatial transcriptomics spots as a scale (each spot is 55 μm in diameter, and spaced 100 μm center-to-center), we selected < 200 μm circular regions encompassing the device tract and the center point as the “near” region. For the “far” region, a concentric circle at 500 μm from the center using the visium spots was selected. To ensure consistency in differential expression, the same number of spots were selected for near and far regions in each sample (Supplementary Table 8). Specifically, for 1-week and 6-week samples near regions, the average number of spots selected was 19 ± 0 (mean ± SEM). For 1-week and 6-week samples far regions, the average number of spots selected were 20 ± 0 (mean ± SEM).

Assessments of various areas in the tissue samples were carried out between sections as well as within tissue sections at each timepoint. Groups, or clusters, were generated by manually selecting regions in tissue sections for standard differential expression analysis via LoupeBrowser (10x Genomics). Significantly upregulated and/or downregulated genes were found in lists of DEGs generated for: (a) near implant site ( < 200 μm) comparisons between 1 and 6-week tissue (Supplementary Table 1); (b) near ( < 200 μm) implant vs. far (∼500 μm) from implant comparisons within 1-week (Supplementary Table 2), and 6-week (Supplementary Table 3) tissue samples; and (c) far implant site (∼500 μm) comparisons between 1 and 6-week tissue (Supplementary Figure 1 and Supplementary Table 9). Here, near implant site regions reflect local electrode effects while far from implant site regions reflect distal electrode effects. Specific significant DEGs are discussed based on publicly available gene databases (GeneCards., 2025; NIH, 2025), and previously known associations of cellular expression and interactions.

#### 2.3.2 Trial of methods to correct batch effects

High-dimensional transcriptomic data produced by spatial transcriptomics platforms like Visium (10x Genomics) can be prone to batch effects arising from both technical and biological sources (e.g., variability related to unique slides, experimenter differences, assay processing, and sequencing runs). Before using the raw data for downstream analysis, we explored three methods of batch correction to address potential technical artifacts across samples and Visium (10x Genomics) assay runs: (a) Seurat integration (Hao et al., 2024), (b) Harmony (Korsunsky et al., 2019), and (c) Linear Models for Microarray Data (Limma) (Ritchie et al., 2015). Seurat’s integration pipeline produces an integrated assay that does not represent true gene expression values, and instead presents corrected latent representations optimized for alignment across datasets. This integrated data is suitable only for clustering and visualization—not for differential or co-expression analyses. Similarly, Harmony performs batch correction entirely in low-dimensional latent space by adjusting principal component analysis (PCA) embeddings, not gene expression values. This aligns the data structure for clustering, visualization and trajectory analysis instead of reconstructing corrected gene-level expression for differential or co-expression analysis. Contrarily, Limma produces expression matrices that are directly usable for downstream differential or co-expression analysis. However, the linear nature of correction assumes normal distribution of RNA-seq data which may be problematic for unbalanced, non-linear designs (i.e., high dimensional spatial transcriptomics data) and data with high sample variability. The corrected, homogenous Limma output would need additional testing, transformation and inspection to ensure accurate results in downstream analyses. Using these pipelines and common visualization techniques employing dimensional reduction (Uniform Manifold Approximation and Projection (UMAP), we noted apparent clustering of our data related to slide number, time point, and operator. Presumably, time point clustering is reflective of true biological variation due to the duration of implantation. Slide ID clustering was improved with all 3 pipelines, although each method carries limitations that undermine downstream analysis, as noted above. All observations and associated discussion are summarized in Supplementary File 1. Thus, none of these three approaches offered the desired combination of batch correction and alignment with our downstream data analysis needs.

We also plotted our data after aggregation with the standard SpaceRanger pipeline, and we visualized the results in UMAP, parsed by operator, slide ID, and time point (see Supplementary File 1) in Loupe Browser (10x Genomics). We observed minimal clustering for slide ID and time point, and apparent clustering for operator. When considering the broader limitations and complications of the tested pipelines, we proceeded with the standard differential expression analysis pipeline using LoupeBrowser for the following reasons: (a) within each sample, the number of spots selected for differential expression analysis was relatively low (near and far regions, ∼540 spots in total) when compared to all mapped spots (∼32,900 spots) that are plotted on UMAPs to visualize clustering patterns—of these selected spots, an even smaller number (∼78 spots) are included from the “Operator 1”-affected samples, (b) Loupe Browser employs correction and normalization of the data prior to differential expression analysis, and (c) this pipeline outweighed the others in terms of ease and efficiency in generating results.

#### 2.3.3 Variance calculation

Similar to the spot selection mentioned in section 2.3.1, a custom-made MATLAB script allowed the user to select a center point of tissue injury using qualitative observations of spatial transcriptomics *Gfap* gene expression distribution, and comparison with regions selected in LoupeBrowser. An automated selection of the near and far regions using the same criteria (e.g., 200 μm region around the center point as near region, and a concentric circle of spots at 500 μm from center point as far region) was done for each of the 14 samples. The area of spot selection was kept consistent, ensuring that similar number of spots were selected in each region (Supplementary Table 8). Specifically, for 1 and 6-week samples near regions, the selected areas contained 18.71 ± 0.64 spots and 17.86 ± 1.22 spots (reported as mean ± SEM), respectively. For 1 and 6-week samples far regions, the respectively selected areas contained 20.14 ± 0.40 spots and 19.29 ± 1.06 spots (reported as mean ± SEM). We generated normalized “pseudobulk” count matrices for both the near and far sites. Median-normalized average (MNA) was computed using raw gene counts extracted from the Space Ranger raw feature and barcode matrix. To account for spot-to-spot differences in sequencing depth, and number of spots, the median-based scaling was conducted as follows (in accordance with the standard Visium pipeline). For each spot *j*, a size factor *s*_*j*_ was defined as the following:


$$s_{j}=\frac{T_{j}}{\mathrm{median}\,\left(T\right)}$$

where T_j_ = ∑_g_ c_g,j_ means the total gene expression counts in spot *j*, and *c*_*g,j*_ represents the raw counts of genes *g* in that spot. Normalized values were calculated as


$${\tilde{c}}_{g,j}=\frac{c_{g,j}}{s_{j}}.$$

For each gene *g*, the MNA value was defined as the arithmetic mean of normalized expression across all *N* spots within the near region:


$${\mathrm{MNA}}_{g}=\frac{1}{N}\,\overset{N}{\underset{j=1}{\sum }}{\tilde{c}}_{g,j}.$$

Once the MNA was calculated for each gene, we investigated the variation in normalized counts between samples to explore the variability in the expression and spatial distribution of device-induced DEGs (Supplementary Table 4). We focused our analysis on the top DEGs, in terms of LFC, for the 1- and 6-week time points, and calculated the variance of normalized counts between samples within each time point. We plotted the spatial landscape of these genes to assess the variation in the spatial footprint of DEGs between samples. Scatter plots and bar graphs to visualize DEG variance across samples were created using GraphPad Prism 11.0.0 for Windows (GraphPad Software, Boston, Massachusetts, United States).

#### 2.3.4 Cell type-specific association of DEGs

The Visium assay used in this study has a key benefit of being a whole-transcriptome assay, appropriate for a wide search of potential device-induced effects. However, it has a supracellular spatial resolution (assessing gene expression within 55 μm diameter domains), and thus, DEGs cannot be assessed on a cell type-specific basis. We employed a factorization strategy, non-negative matrix factorization (NMF), based on previously reported methods (Riggins et al., 2023), to decompose normalized spatial transcriptomic data into spatial cell-type abundance maps and corresponding expression profiles. In essence, this produces a correlative association between cell type-specific markers and gene expression: if a specific gene is especially abundant in spots dominated by microglial-associated genes, then we can infer that microglia are likely to be the primary source of the expression of that gene. The cell type categories were optimized using an Alternating Direction of Multipliers Method (ADMM)-based algorithm, as previously described (Riggins et al., 2023). Best model ranks were determined empirically, with the major glial types (astrocytes, microglia, and oligodendrocytes) associated with a single class, whereas neurons were best represented by 2 subtypes (putative excitatory and inhibitory neurons). We used gene lists associated with 4 major cell types in the central nervous system, (neurons, astrocytes, microglia, and oligodendrocyte precursor cells, OPCs), supplemented with GFAP to identify astrocytes (Zhang et al., 2014). We focused on these 4 cell types as having predominant relationships with device-induced reactivity genes (in particular, astrocytes and microglia). Of note, the opportunity to further subclassify and add other cell types to the analysis, including mature oligodendrocytes, exists for future studies. We used these genes lists provide an initial assessment of the relationship between reactivity genes and individual brain cell types in our data.

Visium data was normalized in Seurat using SCTransform, and gene-spot count matrices were imported into MATLAB for low-rank NMF. To quantify the association between cell type-specific spatial maps and reactivity gene distributions, we have used two complementary metrics. First, Pearson correlation coefficients were calculated between each NMF spatial factor and each reactivity gene, following a square-root transformation of both vectors to stabilize variance in the count data. Second, cosine similarity was computed as cos(θ) = (u ⋅ v)/(||u|| ||v||), where u and v represent the NMF spatial factor and reactivity gene distribution as vectors across all spatial spots, and |⋅| denotes the Euclidean norm. Values range from 0 (no spatial overlap) to 1 (identical spatial distribution), as NMF constrains all factor values to be non-negative. Statistical significance for cosine similarity was assessed empirically using 10E5 random permutations, where p-values reflect the proportion of random simulations exceeding the observed overlap. Both metrics were computed for all pairwise combinations of NMF factors (Astrocyte, Neuron 1, Neuron 2, OPC, and Microglia) and reactivity genes (Supplementary Tables 5, 6). Additionally, a joint NMF analysis was performed by pooling all spatial spots across samples within each time point (1 and 6-week) and running a group-level factorization to identify spatial patterns common across all samples within each time point (Supplementary Table 7).

#### 2.3.5 DiffCoRank pipeline

While DEGs are informative for understanding the association between single genes and device-induced effects, we are likewise interested in the associations between genes as a more holistic view of device-tissue interactions. We applied a differential co-expression method recently reported by our lab, “DiffCoRank,” to identify associated effects (Chakraborty et al., 2025). The method is based on a previously described approach (“DiffCoEx”) (Tesson et al., 2010) and allows identification of modules of genes associated with implant-driven tissue responses. Modules are defined based on similarity in device-induced changes in correlation of expression across samples between near vs. far regions relative to the implant for 1- and/or 6-week samples. Further, genes central to each module (hub genes) can be identified as key biomarkers associated with each gene group.

Here, differential co-expression analysis was performed using the DiffCoRank framework (Chakraborty et al., 2025) on pseudobulk spatial transcriptomic data generated from the selected regions of all 14 samples as mentioned in section 2.3.3. Three comparisons were carried out: (a) near region relative to the implant at 1- vs. 6-week, (b) near vs. far regions relative to the implant for 1-week samples, and (c) near vs. far regions relative to the implant for 6-week samples. Data were first filtered to focus on reliably expressed (raw count ≥ 100), protein-coding genes. Gene modules were identified with a combination of UMAP and density-based spatial clustering of applications with noise (DBSCAN), and multiple network-based criteria were used identify hub genes (degree, closeness, betweenness, and eigenvector centrality), as previously described (Chakraborty et al., 2025).

## 3 Results

### 3.1 Differentially expressed genes (DEGs) between and within 1 and 6-week implants

**FIGURE 1:**
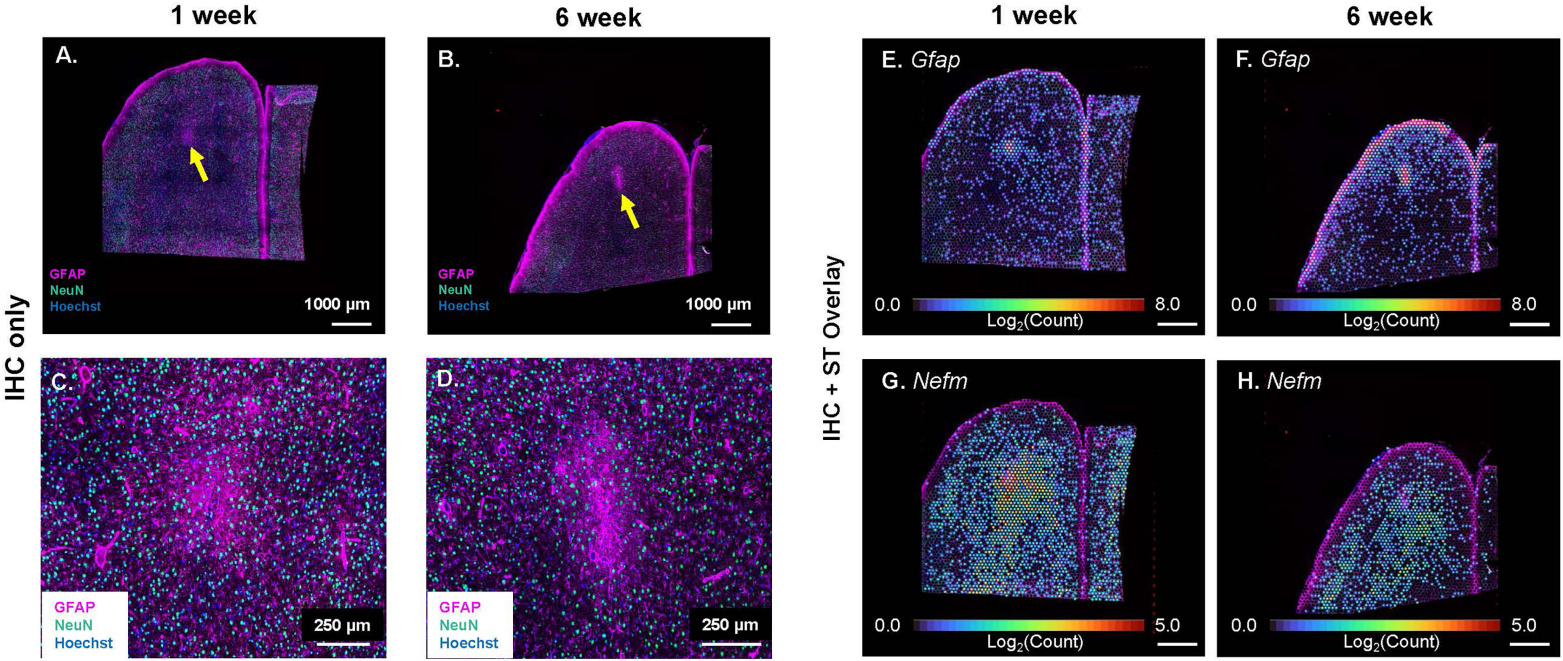
Immunohistochemistry and spatial transcriptomics of conventional markers of the tissue response around brains implanted electrodes for 1-week and 6-weeks. Representative **(A)** 1-week and **(B)** 6-week tissue sections immunostained for glial fibrillary protein (GFAP: magenta), neuronal nuclei (NeuN: green) and Hoechst (universal nuclei counterstain: blue). Upon qualitative observation, GFAP is highly expressed near at the implant site at both time points, and NeuN expression decreases at 6-weeks. **(C,D)** Closer view of the respective injury site. Representative spatial transcriptomics data overlaid on IHC images to show differential gene expression of *Gfap* and *Nefm* at **(E,G)**. 1-week and **(F,H)** 6-week implanted tissue. Scale bars are 1,000 μm in **(C–F)**.

Representative wide-field histological images of 1- and 6-week tissue sections immunostained for GFAP, NeuN and Hoechst are shown in Figures 1A,B, with the bottom panel offering a magnified view of the respective implant site or device tract (Figures 1C,D). By qualitative observation, the expression of GFAP, a commonly used glial protein marker to assess tissue response at the device-tissue interface, is increased around and at the implant site in tissue sections of both time points. The overlay of spatial transcriptomics on wide-field-stained images (Figures 1E–H) can offer insight into the gene expression patterns of similar conventionally used markers. As shown, *Gfap* differential gene expression at the 1-week time point is upregulated at the implant site, and somewhat spread out within a close (∼200 μm) radius surrounding the injury. At the 6-week time point, the *Gfap* gene expression appears more consolidated, indicative of glial encapsulation of the device. Gene expression of neurofilament medium chain (*Nefm*), a protein encoding gene for neurofilament that is involved in maintaining neuronal structure integrity, is upregulated near and around the device tract at 1-week. Increased expression of *Nefm* has been related to neuronal damage (Salatino et al., 2017b) suggesting prominent initial neuron injury at the 1-week tissue. While at 6 weeks, *Nefm* gene expression appears to decrease, supporting previously reported decreases in neuronal density and neuron function genes near chronic implants.

**FIGURE 2:**
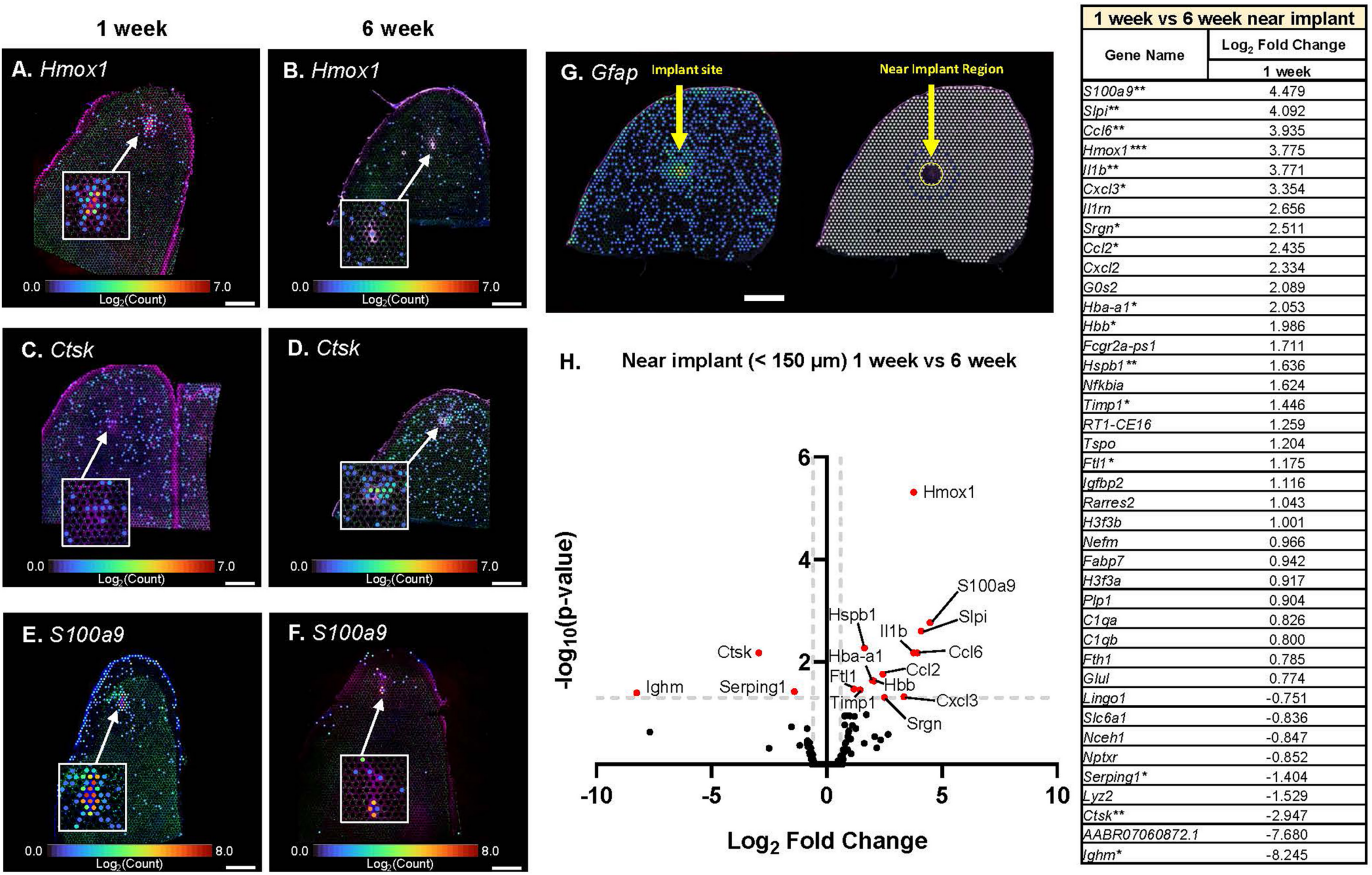
Differentially expressed genes (DEGs) near the 6-week implant site relative to near the 1-week implant site (within 200 μm of implant site). **(A,C,E)** Visium gene expression heatmaps of specific genes differentially expressed at the 1-week tissue section compared to the same genes differentially expressed in **(B,D,F)** 6-week tissue sections. **(G)** Near sites were manually selected for each tissue section based on known gene expressions (*Gfap*) indicating implant site location. These sites were kept consistent as the immediate ∼150–200 μm region surrounding the device tract. For all near samples, the average number of spots selected in the region was 19 ± 0 spots (mean ± SEM). **(H)** All DEGs represented on volcano plot, and top 40 DEGS are listed in the table. Significance *p* < 0.05*, < 0.01**, 0.001***. Total *n* = 10 rats (*n* = 7 rats per time point). Scale bars are 1,000 μm.

Differential expression analysis of near implant sites ( < 200 μm, manually selected as shown in Figure 2G) between 1 and 6-week tissue samples revealed 16 significant DEGs (*p*adj*-*value < 0.05, Supplementary Table 1). Manual selections of spatial regions can be facilitated by analyzing spatial patterns of certain genes or known markers of pro-inflammatory astrocytes, macrophages and microglia to implanted electrodes, such as *Gfap* and *Gpnmb*, in LoupeBrowser (10x genomics). Figures 2A–F show spatial gene expression overlaid on IHC images for three significant DEGs: *Hmox1*, *S100a9*, and *Ctsk*. Heme oxygenase 1 (*Hmox1*; LFC: +3.78, *p*-value: 4.87E−06) has been associated as a regulator of the macrophage/microglia system during neuroinflammation and injury (Xuan et al., 2023; Yeudall et al., 2024). Here, significant differential expression of *Hmox1* at the 1-week timepoint compared to the 6-week timepoint supports initial injury potential dominance of the microglial cell type near the device. A previous study identified *Hmox1* as a ferroptosis hub gene associated with M1 type microglia/macrophage polarization in spinal cord injury, suggesting a functional shift of macrophages in response to the inflammatory environment (Xuan et al., 2023). Similarly, S100 calcium-binding protein A9 (*S100a9*; LFC: +4.48, *p*-value: 1.71E−03), which is highly expressed in neutrophils, has also been implicated in M2 macrophage polarization (Mizutani et al., 2023). Members of the S100 family of proteins, such as *S100a9*, *S100a8*, and *S100a12*, have been recognized for roles in microbial resistance and immune homeostasis maintenance (Denstaedt et al., 2018; Xia et al., 2024). Our findings show similar significant levels of *S100a9* and Secretory Leukocyte Protease Inhibitor (*Slpi*; LFC: +4.09, *p*-value: 2.49E−3) expression (Figure 2H) suggesting an abundance of myeloid lineage cells and a probable interaction to regulate the inflammatory response near the device at 1-week; *S100a9* drives inflammation, induces cytokine expression and recruitment of neutrophils (Denstaedt et al., 2018), while *Slpi* counterbalances by inhibiting pro-inflammatory mediators and macrophage responses (Tyshchenko et al., 2025). Meanwhile, deficiency of Cathepsin K (*Ctsk*; LFC: −2.95, *p*-value: 6.65E−03) has been linked to immature astrocytes and altered levels of cyclic nucleotide phosphodiesterase in oligodendrocytes in the mouse hippocampus (Dauth et al., 2011). Our findings show significant downregulation of *Ctsk* near the device at 1-week, pointing to heightened cellular structure and metabolic changes at this earlier timepoint, potentially pronounced in astroglial and oligodendroglial cell types. Significant downregulation of Immunoglobulin heavy chain constant mu (*Ighm*; LFC:−8.25, *p*-value: 4.01E−2) near the device at 1 week could be linked to previous reports uncovering dramatically decreased *Ighm* expression in microglia following lipopolysaccharide (LPS) stimulation (Morimoto et al., 2025), suggesting an increased response to inflammation in the earlier stages of tissue injury. Serpin Family G member 1 (*Serping1*; 1.40, *p*-value: 3.76E−2) is decreased near the device at 1 week compared to near the device at 6 weeks, presenting potentially less neurotoxic reactive astrocytes in the near implant regions of 1-week samples (Liddelow et al., 2017).

**FIGURE 3:**
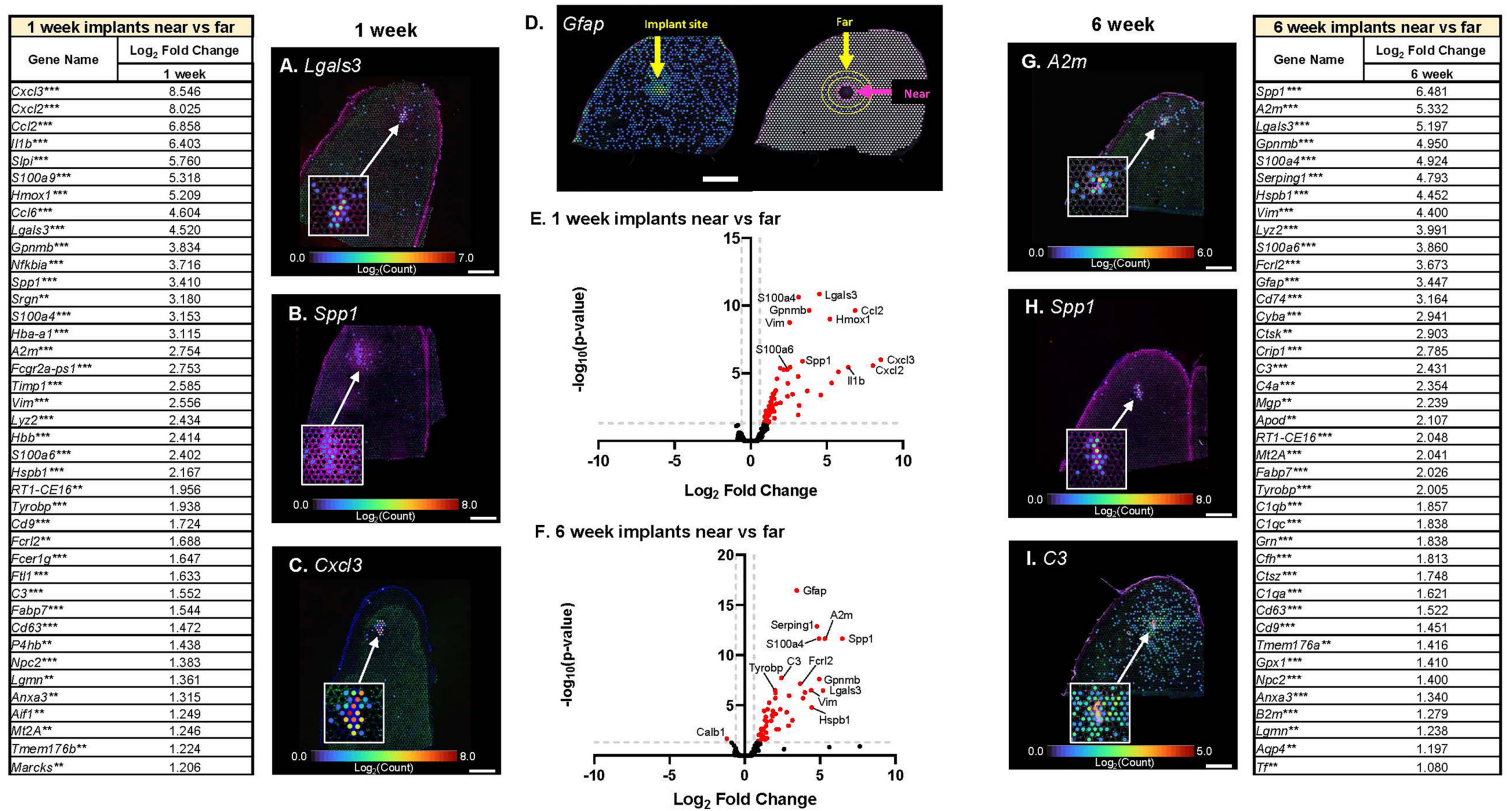
Differentially expressed genes (DEGs) near relative to far from the implant for 1-week implanted tissue and 6-week implanted tissue. Spatial transcriptomics revealing highly upregulated genes near vs far regions in **(A–C)** 1-week implanted tissue, and **(G–I)** 6-week implanted tissue. **(D)** Respresentative image of manually selected near vs far regions in a spatial transcriptomics tissue section. Near implant sites were kept consistent as the immediate ∼150–200 μm region surrounding the device tract, while far site regions (∼500 μm) were selected to surround and remain within a < 2 mm radius of the device tract to minimize whole-tissue effects that may include cellular processes prominent in other brain regions. For 1-week and 6-week samples near regions, the average number of selected spots were 19 ± 0 spots (mean ± SEM). For 1-week and 6-week samples far regions, the average number of spots selected were 20 ± 0 spots (mean ± SEM). **(E, F)** Volcano plots of all DEGs for near vs far regions in 1-week and 6-week implants. Lists of top 40 DEGs for each time point are shown in tables. Significance *p*-value < 0.05*, < 0.01**, 0.001***. Total *n* = 10 rats (*n* = 7 rats per time point). Scale bars are 1000 μm.

Within section comparisons across 1-week and 6-week samples provide further insight into DEGs near ( < 200 μm) and far (∼500 μm) from the implant site (Figure 3D) for each time point, revealing: (1) 63 significant DEGs (*p*adj-value < 0.05, Supplementary Table 2) in 1-week implants; and (2) 57 significant DEGs (*p*adj-value < 0.05, Supplementary Table 3) in 6-week implants. Significant upregulation of Secreted Phosphoprotein1 (*Spp1*; LFC +3.41, *p*-value:1.28E−06) is prominent near the device compared to far regions in 1-week implants (Figure 3B). *Spp1*-expressing microglia have been reported as resembling disease-associated microglia (DAM-like microglia) that have been identified as immune sensors of neurodegeneration (Deczkowska et al., 2018; Lan et al., 2024). Our findings show higher upregulated expression levels for *Spp1* (LFC +6.48, *p*-value: 2.26E−12) at 6-weeks near the device compared to far from the device (Figure 3H), suggesting higher expression of the gene overall by microglia and other cell types, or a greater number of cells expressing *Spp1* consolidated near the device tract at the later time point compared to 1 week. Significant upregulation of an apoptosis regulating gene encoding for Galectin-3 (Perillo et al., 1995) (*Lgals*; LFC: +4.52, *p*-value: 1.36E−11 at 1-week, and LFC: +5.20, *p*-value: 3.29E−07 at 6-weeks) is evident near the device within both 1-week (Figure 3A) and 6-week implants. *Lgals3* is involved in the innate immune response as well as inhibition of apoptosis, and abnormal expression of *Lgals3* is associated with certain cancers and inflammatory diseases (Farhad et al., 2018; Li et al., 2021). Similarly, *S100a4* (and other *S100a* family genes), encoding for calcium-binding protein A4 is also involved in regulating apoptosis, and irregular expressions of this gene have been related to cancer (Bresnick et al., 2015). *S100a4* is significantly upregulated near the device site within 1-week and 6-week samples (*S100a4*; LFC: +3.15, *p*-value: 2.31E−11 at 1-week, and LFC: +4.92, *p*-value: 2.261E−12 at 6-weeks). Complement factor 3 (*C3*; LFC: +1.55, *p*-value: 3.25E−4 at 1-week, and LFC: +2.43, *p*-value: 1.871E−08 at 6-weeks) is a central protein of the immune system’s complement cascade, and is upregulated in both 1-week and 6-week samples (Figure 3I), marking maintained innate immune response across the two time points (Kuska et al., 2025). Another immune-system related gene encoding for Lysozyme 2 (*Lyz2*; LFC: +2.43, *p*-value: 5.26E−05 at 1 week), crucial for microbial defense and innate immune response (Ferraboschi et al., 2021), is upregulated near the implant compared to far regions in 1-week tissue sections as well as within 6-week tissue sections (*Lyz2*; LFC +3.99, *p*-value: 5.24E−07). Significant upregulation of Glycoprotein NMB encoding gene (*Gpnmb*) is present near implant sites within 1-week (LFC: 3.83, *p*-value: 2.30E−10) and 6-week (LFC: 4.95, *p*-value: 2.46E−08) implants, specifying immune system regulation. *Gpnmb* overexpression has also been linked to various cancers, potentially playing an immunosuppressive role (Lazaratos et al., 2022). Together, these findings support that expression levels of some genes are maintained to a certain degree across the early 1-week and chronic 6-week time period.

Interestingly, and as noted previously, a subset of genes encoding for chemokines are highly upregulated near the device at 1 week compared to far regions, including *Cxcl3* (Figure 3C, LFC: +8.55, *p*-value: 9.93E−07), *Cxcl2* (LFC: +8.03, *p*-value: 2.67E−06), *Ccl2* (LFC: +6.86, *p*-value: 2.30E−10), and *Il1b* (LFC: +6.40, *p*-value: 3.64E−06), that are not significantly differentially expressed in the 6-weeks samples. Contrarily, absent in 1-week samples, Alpha-2 macroglobulin (Figure 3G, *A2m*; LFC: +5.33, *p*-value: 2.26E−12) is highly upregulated near the device at 6 weeks, and has been reported as a marker of neuronal injury in Alzheimer’s disease (AD) (Varma et al., 2017). Reduced Calbindin-1 (*Calb1*; LFC: −1.20, *p*-value: 2.12E−2), the only significantly downregulated DEG revealed at 6-weeks, has also been observed in AD human and mice brains, suggesting its critical role in AD pathogenesis and cognitive disorders (Datta et al., 2024; Kook et al., 2014). As the top significantly DEG near the implant tract compared to far regions in 6-week samples, *Gfap* (LFC: +3.45, *p*-value: 8.68E−17) is indicative of previously recognized chronic glial encapsulation of the device (Salatino et al., 2017a).

### 3.2 Variance of top differentially expressed genes (DEGs) near the device across samples

**FIGURE 4:**
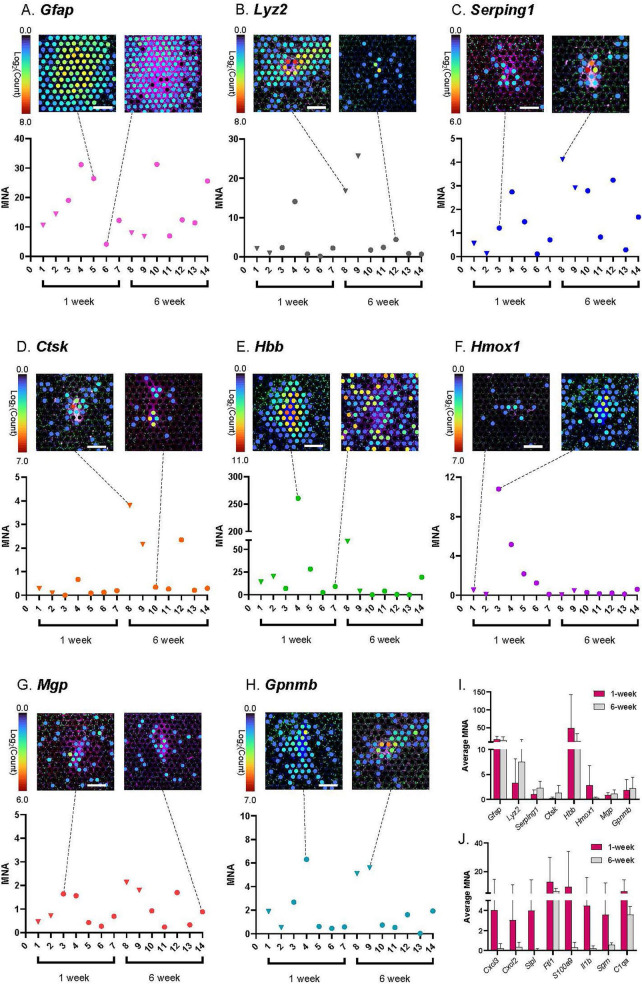
Variability of median normalized averages (MNA) of top DEGs in the near region of the implanted interface across samples. Sample-to-sample variances of DEGs provide insight into the cellular and molecular landscape of the foreign body reaction to devices at 1-week and 6-weeks. Scatter plots **(A–H)** present MNA values for 1-week and 6-week samples for the selected DEG. Triangular spots indicate samples collected by experimenter 1 while circular spots indicate sampled collected by experimenter 2. Heat maps above are shown for the traced sample points on the scatter plot to demonstrate spot intensity differences in high or low variability of the gene for a given time point or across time points. Bar graphs summarize overall variabilities by plotting average MNA values to **(I,J)** average MNA values for DEGs between 1 week and 6 week implants. Error bars indicate standard deviation across samples.

Sample-to-sample variation in our large spatial transcriptomics dataset not only affects overall differential expression analysis but also provides insight into the cellular and molecular landscape of the FBR to implanted electrodes over the 6-week period. Investigations of most of the individual top significant DEGs, discovered in the section above, were carried out for each sample. The median normalized average (MNA) counts were calculated (Supplementary Table 4) for a selected set of highly DEGs in the region of interest (near the device tract < 200 μm radius) in each sample; some of these are plotted for comparison and visualization (Figure 4).

Higher sample-to-sample expression variance is observed across 1-week samples compared to 6-week samples (Supplementary Table 4). At 1 week in comparison to 6 weeks, fewer DEGs exhibit relatively low variance, suggesting a more heterogeneous and dynamic cellular and molecular landscape at the earlier time point. Conversely, while most genes exhibit low sample-to-sample variability at 6 weeks, a few highly expressed genes, including *Gfap*, *Lyz2, Clu*, *Serping1*, *Ctsk*, and *Ighm* display relatively increased MNA values along with higher sample-to-sample variance. These patterns correspond with DE results reporting significant upregulation of these genes near the device at this later time point.

Notably, a subset of genes maintains similarly low variability across both time points, including *Cyba*, *Cfh*, *Tmem176a*, *Mgp*, *Cnn3*, and *Ctsh*, while others—such as *Gpnmb*, *C3*, *Apod*, and *Tf*—exhibit higher, yet comparable, variance levels at both 1 week and 6 weeks. An additional key observation is that at 1-week, multiple DEGs including—*Cxcl3*, *Spp1*, *Il1b*, Cxcl2, *Slpi*, *Hba-a1*, *S100a9*, *Ftl1*, *Hbb*, *C1qa*, *B2m*, and *Sgrn*—demonstrate markedly high variance across samples (Supplementary Table 4). By 6 weeks, however, only three of these genes (*Hba-a1*, *Gfap*, and *Hbb*) maintain high variance values, indicating a time-dependent narrowing of transcriptional heterogeneity as the tissue response transitions toward a more stabilized chronic state.

Generally, by 6 weeks, the variability of the investigated DEGs across samples is decreased. This supports previous reports on the nature of acute to chronic FBR progression, marking a more stable and less widespread reaction at the interface at later time points (Gupta et al., 2024; Salatino et al., 2017a; Whitsitt et al., 2021; Whitsitt et al., 2024). In our results, certain gene variances are inversely related, such as—*Cxcl3*, *Cxcl2*, *Il1b*, *Slpi*, *S1009*, *C1qa*, and *Sgrn*—across the two time points; all with higher variances at 1 week, and lower at 6 weeks. A lone sample in the 1-week cohort drove the markedly high variance for many of these genes; however, this effect is also notable in the 6-week cohort. At 6 weeks, one sample had higher MNA values for the maintained high variance genes (i.e., *Hba-a1* and *Hbb*) compared to other samples in the cohort. The trend of a particular sample in an implanted cohort contributing to high variability of certain genes suggests that an aggravated FBR in the given sample, potentially owing to heightened insertional damage or other variables, can drive LFC responses in the overall data set.

### 3.3 Non-negative matrix factorization (NMF) to identify cell type contributors to the device-tissue interface

**FIGURE 5:**
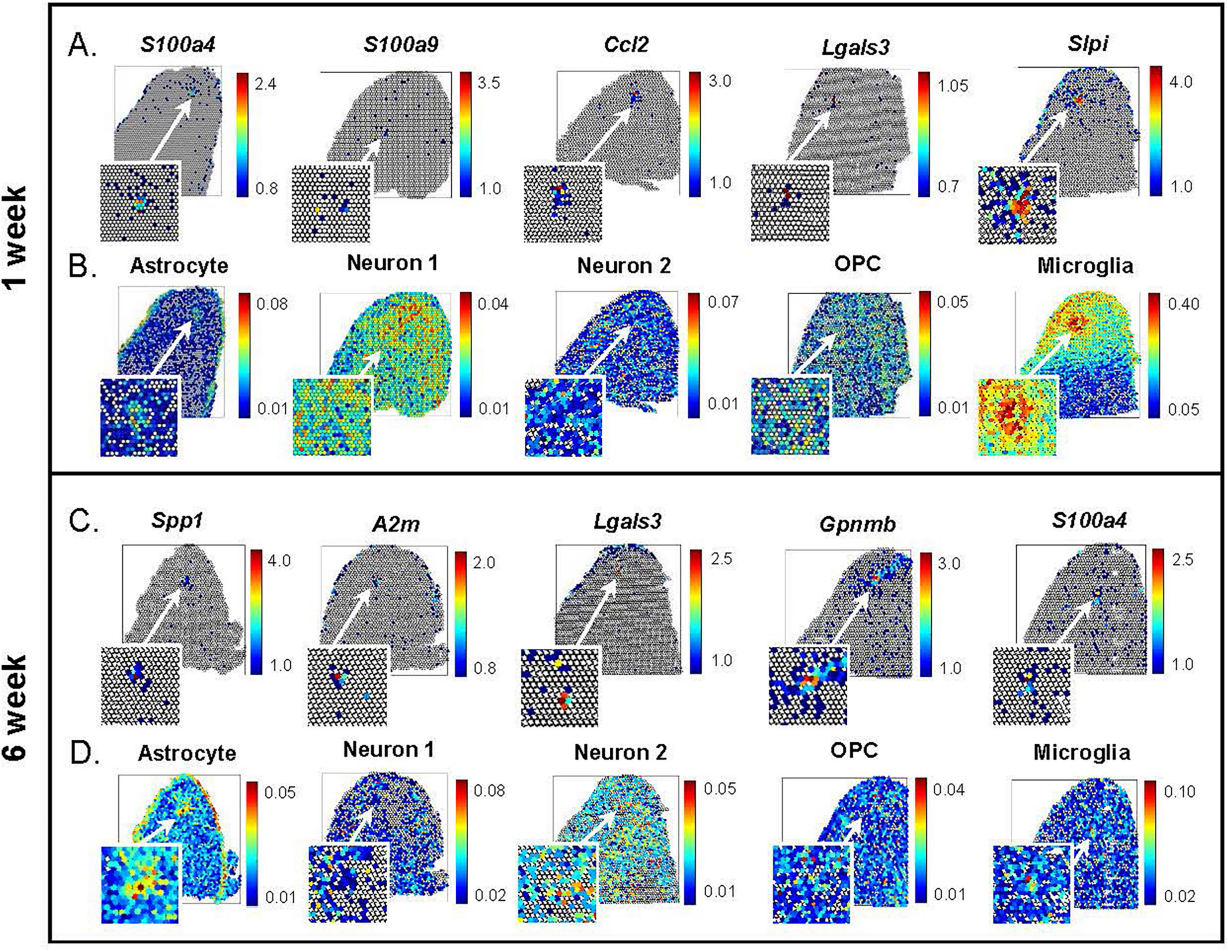
Representations of spatial distribution of top DEGs and non-negative matrix factorization (NMF) of cell types across the device-tissue interface for **(A, B)** 1-week (top panel) and **(C,D)** 6-week (bottom panel) samples. In each panel, top row represents example tissue sections of the given time point of top reactivity genes, and bottom row contains corresponding spatial representations of different cell type contributors from NMF analysis.

We used a factorization approach to infer the predominant cellular contributor(s) to the most highly DEGs surrounding devices at both timepoints (Figure 5). Generally, at 1-week, NMF indicated that microglia and astrocytes were the predominant contributors of each of the most highly DEGs (Supplementary Tables 5, 7). At 1-week, microglia were the dominant contributor, although in most samples, astrocytes returned a cosine value which was slightly lesser, but very similar in magnitude to those returned by microglia. A few of the samples (∼40%), returned higher cosine values for microglia across many of the highly DEGs, such as *Cxcl3*, *Cxcl2*, *Il1b*, *Slpi*, *Hmox1* and *Spp1*, suggesting that the inflammatory glial reaction to the implant was driven primarily by reactive microglia in these samples. At 6 weeks, the predominant cellular contributors to the most highly DEGs were also mixed between microglia and astrocytes, but with increased consistency across samples (Supplementary Tables 6, 7). Astrocytes were the dominant source of *A2M*, *S100a4*, *Serping1*, *Vim*, *Lyz2*, and *S100a6*, while microglia were the dominant source of the remaining highly DEGs. Interestingly, NMF revealed the emergence of a negative correlation of many of these genes with neurons and oligodendrocytes at the 6-week time point, which was also evident in certain samples at the 1-week time point: in particular, *Spp1*, *A2M*, *S100a4*, *Serping1*, *S100a6*, *Slpi*, *Il1b*, and *Cxcl3* all displayed inverse spatial association with all 3 cell types (putative excitatory neurons, putative interneurons, and oligodendrocytes). Taken together, the NMF results imply initial microglial and astroglia activation followed by an increasingly inflammatory state in astrocytes, coupled to the emergence of negative impacts on neuronal and supportive oligodendrocyte cells by the 6-week time point. Furthermore, at the 1-week time point, intermittent and elevated cosine values for microglia and astrocytes across samples—rather than a uniform pattern—point to greater variability in cell-type contributions.

### 3.4 DiffCoRank to uncover hub genes and differential co-expression of genes near to and far from the device

**FIGURE 6:**
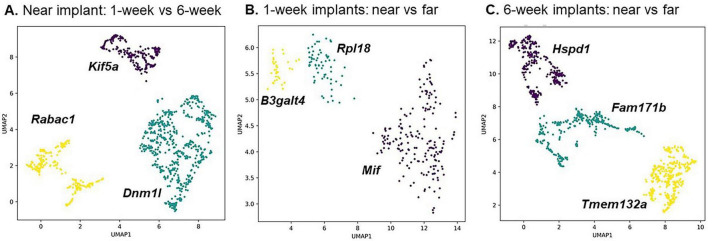
Modules and hub genes displayed in uniform manifold approximation and projections (UMAPs), identified using DiffCoRank for three conditions: **(A)** near implant regions in 1-week vs 6-week samples, **(B)** near vs far region relative to the implant for only 1-week samples, and **(C)** near vs far regions relative to implant for only 6-week samples. Gene modules represent closely related genes, and hub genes, or markers of central role, of each module are labeled. Conditions included RNA-seq data from rat brain tissue implanted with devices for 1-week (*n* = 7 rats) and and 6-weeks (*n* = 7 rats).

Analysis of differentially co-expressed gene clusters in the Visium (10x Genomics) dataset reported here could provide further insight into unique regulatory processes and consistent co-expression patterns at the device interface. We implemented the comprehensive framework of DiffCoRank, previously reported by our lab (Chakraborty et al., 2025), to assess gene expression in three conditions: (1) near regions to implant ( < 200 μm) for 1-week vs. 6-week samples, (2) near vs far (∼ 500 μm) regions relative to the implant site for only 1-week samples, and (3) near vs. far regions relative to the implant site for only 6-week samples. These assessments provided us with groups of genes, or modules, with distinct correlation patterns across samples in each condition. For each module, “hub” genes, or genes hypothesized to have a central role in the module, were identified to present markers that best represent the essential regulatory processes occurring in the tissue for the given comparison. In the near regions relative to the implant for 1- and 6-week samples, three hub genes were identified, namely *Kif5a*, *Rabac1*, and *Dnm1l*. Kinesin family member 5 A (*Kif5a*) is a neuron-specific marker, regulating neuronal health and function via anterograde axonal transport, and guiding axonal regrowth post injury (Guerra San Juan et al., 2025). Mutations in *Kif5a* have been linked with many motor neuron and neurodegenerative diseases (Cozzi et al., 2025). Likewise, Rab Acceptor 1 (*Rabac1*) interacts with the Rab family of GTPases that are essential for vesicular trafficking, synaptic architecture and function, and could be implicated in Parkinson’s disease mechanisms due to disrupted trafficking pathways (Gitler et al., 2008). Dynamin-1 like (*Dnm1l*) is involved in regulating mitochondrial fission, mitosis, and overall brain development (Magistrati et al., 2025). The gene module for *Dnm1l* (Figure 6A) is the largest in size, indicating that most genes across 1-week and 6-week samples were involved in developmental and metabolic processes. Together, these hub genes suggest that the major processes at work near the implant at 1-week and 6-weeks are related to neuronal health, injury rescue and related intracellular functions, such as trafficking and cellular energy metabolism.

The hub genes for the near vs far regions relative to the implant in 1-week samples were: *Mif*, *Rpl18*, and *B3galt4*. Macrophage migration inhibitory factor (*Mif*) is an inflammatory mediator and a multifaceted cytokine, critically controlling inflammation and the immune response. It has been reported to be a promising biomarker in past and new literature, suggesting its major regulatory roles in host immune defense as well as neurological diseases (Grieb et al., 2010; Matejuk et al., 2024; Sumaiya et al., 2022). Ribosomal Protein L18 (*Rpl18*) encodes for ribosomal proteins that are involved in neuronal development and plasticity as well as social stress (Mekiten et al., 2023; Smagin et al., 2016). Beta-1,3-galactosyltransferase-4 (*B3galt-4*) has been reported as key enzyme involved in the biosynthesis of gangliosides, important for maintenance of neuronal function and cellular state changes. Altered regulation of *B3Galt4* have been observed to increase cell vulnerability to toxic stressors and contribute to underlying mechanisms of neurogenerative diseases and certain cancers (Madrid et al., 2018; Sha et al., 2022; Verma and Schneider, 2019). Overall, these results corroborate with our findings of pro-inflammatory cytokine/chemokine signaling as one of the prominent processes at play at the device-tissue interface at the earlier 1-week time point. The prominence of stress induced mechanisms that are implicated in neurological and neurodegenerative diseases are also apparent.

At 6 weeks, three hub genes are uncovered near vs far relative to the implant site: *Hspd1*, *Fam171b*, and *Tmem132*. Heat Shock Proteins (HSPs) are involved in cellular homeostasis under heat shock and stresses (Kang et al., 2019). *Hspd1*, or HSP60, is involved in activating innate immune signaling and stimulating cytokine production as well as apoptosis in target cells (Flohé et al., 2003; Kim et al., 2009). Family with sequence similarity 171, member B (*Fam171b*) is another candidate biomarker in a variety of pathophysiological contexts involving neurodegeneration and cancer progression (Hu et al., 2023; Tran et al., 2021). It has been reported to localize vesicle-like structures and synapses of neurons (Tran et al., 2021). Although under-investigated, limited studies report transmembrane protein 132A (*Tmem132a*) as a regulator of Wnt signaling pathway, important for tissue regeneration, neural connectivity and maintenance (Li and Niswander, 2020). Broadly, these results suggest that at 6 weeks, major regulatory processes involving synaptic functioning, tissue remodeling/regeneration, and inflammation driven apoptosis are at play.

## 4 Discussion

Studying the biological response to implanted electrodes has been an active area of inquiry for decades, with the goal of understanding mechanisms of therapy, side effects, and suboptimal performance over time. It only recently has been possible to multiplex readouts across numerous cellular and molecular pathways of interest using modern transcriptomics and proteomics techniques, while retaining critical spatial information (Gupta et al., 2024; Huff et al., 2026; Joseph et al., 2021; Song et al., 2022; Song et al., 2024b; Thompson et al., 2021; Thompson et al., 2023; Whitsitt et al., 2021; Whitsitt et al., 2024). Nonetheless, a critical limitation of these approaches is their prohibitive cost, which practically constrains sample sizes. Our initial studies in this space included smaller sample sizes (2–3 rats) and incorporated non-implanted, naïve tissue to establish baseline gene expression differences relative to implanted brains (Whitsitt et al., 2021; Whitsitt et al., 2024). The present work instead focuses on acute-to-chronic FBR progression within local and distal regions of implanted tissue across a larger sample set, prioritizing robust transcriptional changes with long-term tissue adaptation. In addition to performing differential expression analysis, the larger sample size allowed us to explore sample-to-sample variability and batch effects, while implementing computational approaches for network analysis and cell type specificity of DEGs.

Differential expression analysis of near regions between 1-week vs 6-week implants revealed subsets of significant DEGs that point to broad biological processes at play, including: neuroprotection, neuroinflammation and neurodegenerative pathologies (*Ccl2*, *Ccl6*, *Il1b*, *Srgn*, *Cxcl3*) (Boraschi et al., 2023; Joly-Amado et al., 2020; Nakagawa et al., 2019; Pozzi et al., 2018; Qian et al., 2024; Qu et al., 2023); oxygen transport (*Hba-a1*, *Hbb*); cellular stress and apoptosis regulation (*Hspb1*) (Singh et al., 2024); cell proliferation and tumor cell survival (*Timp1*) (Hekmat et al., 2013), and; iron metabolism (*Ftl1*) (Remesal et al., 2025). Many of these significantly upregulated DEGs point to cellular and molecular functions involving chemokine/cytokine signaling and microglia-mediated neuroinflammation in the near regions of 1-week implants compared to 6-week implants. Similarly, within sample analyses of near vs far regions of 1-week implants showed a subset of genes encoding for chemokines (*Cxcl3*, *Cxcl2*, *Ccl2*, and *Il1b*), which reinforce that macrophage/microglia mediated neuroinflammation is more prominent in the 1-week samples compared to 6-weeks. Meanwhile, near vs far regions of 6-week implants presented neurodegenerative markers such as *A2m* and *Calb1* that were absent in 1-week samples. In addition to confirmation of initial microgliosis followed by astrogliosis, the chronic tissue response at 6 weeks showed high upregulation of neurodegenerative disease and cancer-related markers, that were not prominent at the acute 1-week time point. The 1-week implants saw a surge in chemokine/cytokine signaling genes and microglia-mediated neuroinflammation. A subset of innate immune response genes sustained significant differential expression levels across the 1-week and 6-week timepoints. These results support genes and cellular signatures commonly reported by previous studies of neuronal injury and the broader tissue response to intracortical microelectrode arrays (Druschel et al., 2024; Eles et al., 2017; Kozai et al., 2016; Michelson et al., 2018; Szymanski et al., 2021; Urdaneta et al., 2023). The trend of numerous upregulated genes, with a near absence of downregulated genes, is notable in the volcano plots visualizing all DEGs near vs far from implant site regions within 1-week (Figure 3E) and 6-weeks (Figure 3F) tissue sections. Contrary to our previous reports, which identified large numbers of significant DEGs surrounding electrodes (Whitsitt et al., 2021; Whitsitt et al., 2022), the analyses presented here revealed fewer DEGs across the conditions examined. Several factors likely contribute to the reduced number of significant DEGs observed in this study, including aggregation across a larger cohort (14 rats), which emphasizes the most reproducible and robust gene expression changes driving overall trends, removal of low count genes from analysis, and comparisons restricted to implanted tissue only.

Our investigations on sample-to-sample variation of top DEGs near the device across animals revealed that analysis of DEGs should include consideration for inter-animal variation in interpretation. Chronic neural recordings from implanted brains have reported signal variabilities and instabilities (Chestek et al., 2009; McCreery et al., 2002; Perge et al., 2013; Prasad and Sanchez, 2012; Seaton et al., 2020). Chestek et al. reported on long term recordings from 3 non-human primates and saw action potential peak amplitude decrease by 37% within 8 weeks from implantation (Chestek et al., 2011). Other studies have seen similar signal decays in implanted rodents and non-human primates (Patel et al., 2023; Perge et al., 2013; Purcell et al., 2009). An important aspect of these prior reports, and relevant to our current findings, is that recorded signal variabilities are not consistent or predictable in nature—some implanted brains experience worse signal loss compared to others, potentially indicative of an exacerbated immune response, differences in insertional damage and severance of blood vessels, and/or underlying sources of subject-to-subject variation. Likewise, our MNA and variance calculations of genes highlight an aggravated FBR in some samples, and present specific markers, namely *Cxcl3*, *Cxcl2*, *Spp1*, *Slpi*, *Ftl1*, *Il1b*, *S1009*, *C1qa*, *Sgrn*, *Hba-a1*, and *Hbb*, that may be susceptible to a variable nature across samples, especially in the acute phase (1-week) of FBR to implanted electrodes. Likewise, while neuronal density and immunohistologically identified glial markers poorly correlate with recording outcomes (Michelson et al., 2018), the markers identified here provide alternative hypotheses for the biomarkers reflective of recording quality.

Characterization of high- and low-variance markers can guide future studies as well as gene prioritization in downstream analyses, particularly with respect to identifying single samples driving overall results (Jeon et al., 2023; Shah et al., 2024; Yuan et al., 2024). Furthermore, variance estimates (MNA values) as reported here can enable assessments of effect sizes and necessary sample sizes for experimental designs with appropriate statistical significance to detect gene-specific differences. While it is difficult to translate traditional power analysis approaches to the statistical analyses typically used in transcriptomics datasets, our results suggest that sample sizes chosen here (*n* = 7) are sufficient to capture statistically significant differential gene expression, identify individual samples driving effects, and reveal genes which are highly versus lowly variable between samples.

Our factorization strategy assessed the relationship between the expression of specific genes and contributions from individual cellular subtypes. In line with previous observations, the results indicated predominant contributions of microglia and astrocytes to the initial inflammatory response, followed by a more robust astroglial component by the 6-week time point. These results were accompanied by the emergence of an inverse relationship with the expression of neuronal and oligodendrocyte cells by the 6-week time point. Albeit correlative in nature, this observation supports the hypothesis that reactive glia are responsible for damage and dysfunction in neurons and oligodendrocytes at the device interface. We previously observed progressive worsening of damage to neuronal structure and function following device implantation between the 1- and 6-week time points (Gregory et al., 2023). Here, we can point to a top list of candidate genes that may underlie these responses and serve as effective biomarkers of biocompatibility: *Spp1*, *Hmox1*, *A2M*, *S100a4*, *Serping1*, *S100a6*, *Slpi*, *Il1b*, and *Cxcl3*. These are top genes that fulfill a set of fundamental criteria: (a) they are highly differentially expressed at the device interface, (b) they are predominantly associated with reactive glia, and (c) they are negatively correlated with neuronal and oligodendrocyte markers. These genes have reported roles in inflammation, ferroptosis, neuronal injury, synaptic loss, and more (Chen et al., 2024; Liddelow et al., 2017; Varma et al., 2017; Zhou et al., 2025). Our findings on these genes and their associations with microglial, neuronal and oligodendrocyte cell-types support recent single nucleus RNA-sequencing studies that have profiled cell-type specific tissue responses to intracortical implants (Huang et al., 2026; Zhao et al., 2025; Zhao et al., 2026). Notably, these studies reported on microglial-astrocytic state transitions during chronic glial remodeling that add context to our NMF findings (e.g., shifts in microglial states characterized by Spp1 and DAM-like microglia (Zhao et al., 2026), neuronal compromise and neuron-astrocyte communication (Zhao et al., 2025)), albeit without the benefit of preserved spatial information offered in our results.

The hub genes revealed by our DiffCoRank framework support our differential expression analysis findings, and provide an added perspective on the regulatory and biological processes governing the tissue response at the device-tissue interface. Mainly, near the implant at both 1- and 6-weeks, hub genes associated with neuronal health, axonal transport, mitochondrial metabolism and vesicular trafficking (*Kif5a*, *Rabac1*, *Dnm1*) suggest continued engagement of neuronal maintenance and injury-response pathways. At 1 week, near-vs-far region comparisons were dominated by inflammatory and stress-related regulators (*Mif*, *Rpl18*, *B3galt-4*), indicating cytokine/chemokine signaling and macrophage/microglia-mediated acute inflammation and stress response. Meanwhile, by 6 weeks, hub genes shifted toward pathways involving synaptic function, tissue remodeling, and inflammation-driven apoptosis (*Hspd1*, *Fam171b*, *Tmem132a*), consistent with a transition from acute inflammation to chronic tissue remodeling and homeostatic regulation. An interesting observation among the gene modules is that at 6 weeks, connectivity and closeness of genes appear tighter in the modules (Figure 6C) compared to the relatively loosely packed gene modules of 1-week (Figure 6B). This is indicative of weaker associations between genes at the earlier time point, once again highlighting a less ordered and potentially unsystematic inflammatory reaction at the acute FBR to implanted electrodes. Nonetheless, the hub genes at both time points provide insight into the major biological processes governing the overall immune response at the device-tissue interface.

## 5 Conclusion

This report analyzes a large dataset (*n* = 14 rat brains) of high-throughput, whole-transcriptome spatial transcriptomics (using Visium, 10x Genomics) of the interface between brain tissue and implanted electrodes. The results herein reinforce known aspects of the inflammatory reaction to implanted electrodes, while suggesting new biomarkers discovered via downstream analysis of the data. With our larger data set, we were able to explore sample-to-sample variations in DEGs that are crucial to contextualize identification of key determinants in differential expression patterns. We visualized sources of batch effects and tested means of correction, but we found that our standard pipeline was optimal amongst the pipeline tested. Nevertheless, these potential sources of variation are important to consider during analysis, and further iteration on our approach is warranted in the future. We further implemented recently developed analysis pipelines to investigate prominent cellular contributors and central regulatory processes governing the reaction in spatially defined regions at the acute and chronic time points. Overall, we found that the multifaceted inflammatory response to implanted electrodes was: (a) driven by a greater degree of diversity microglial and astroglial cells in a variable manner, yet underpinned by prominent functional mechanisms dominated by macrophage- and microglial-mediated cytokine/chemokine signaling at 1-week; and, (b) less variable and more coordinated by 6-weeks, as reflected by differential expression of neurodegenerative disease and cancer-related markers, increased astroglial contributions, and the emergence of tightly connected gene networks involving tissue remodeling, apoptosis, and synaptic function. We present a variety of novel markers that could be studied further in the context of device-tissue interactions, inflammation, neural injury, and trauma.

At the same time, certain limitations exist in the results reported here, which should be considered for future experiments and analyses. This dataset can be extended to include a naïve, not-implanted sample (previous reports by our lab present naïve samples collected by Whitsitt et al. (2021), which could present added information on baseline gene expression results and comparisons. Sample variations, while representative of realistic occurrences in clinical and research settings with implanted brains, can majorly influence differential expression analysis of transcriptomics data: some biomarkers of device-tissue interactions are relatively robust across samples, while others may be driven by a minority of samples experiencing heightened reactivity. The latter observation may be due to variations in vascular disruption during device insertion and/or underlying subject-to-subject biological differences. The Visium platform allows for whole-transcriptome mapping which is advantageous for broadly surveying device-induced gene expression changes. However, its supracellular resolution (55 μm diameter spots) results in spatial “hotspots,” with each spot sampling transcripts from multiple cells. With spots spaced 100 μm center-to-center, this design may fail to capture genes from rare cell populations, and lowly expressed transcripts. Single-cell sequencing transcriptomics techniques can address these technical limitations and further the results reported here as well as verify cellular contributors at the device-tissue interface (Thompson et al., 2026).

Overall, we present a workflow to analyze spatially relevant transcriptomics data from multiple samples with a focus on uncovering robust, novel biomarkers determining device-tissue interactions. The biological markers and processes uncovered here could guide future neurological investigations of healthy and pathological brains, especially in the context of neural inflammation and injury. These findings add to the growing understanding of the cellular and molecular landscape at the device-tissue interface, acute-to-chronic FBR progression, and overall immune response to implanted electrodes.

## Data Availability

The data used in this study are publicly available at the following URLs. Previously reported (1 week sample B, 1 week sample C, 6 week sample B and 6 week sample C) can be found at www.ncbi.nlm.nih.gov/sra/PRJNA1089183, and remaining samples are available at https://www.ncbi.nlm.nih.gov/bioproject/1472787. The code used in this study is publicly available on GitHub at https://github.com/msureil/SpatioBiomarker.
