# Supplementary material for: Spatial transcriptomics on an expanded dataset at the brain-electrode interface: exploration of variability and identification of novel biomarkers
Source: Front Neurosci. 2026 Jun 15;20:1852774. doi: 10.3389/fnins.2026.1852774 (PMC13311015; doi:10.3389/fnins.2026.1852774)
Supplement: Supplementary file 2 [file Data_Sheet_2.pdf]

Supplementary Figures

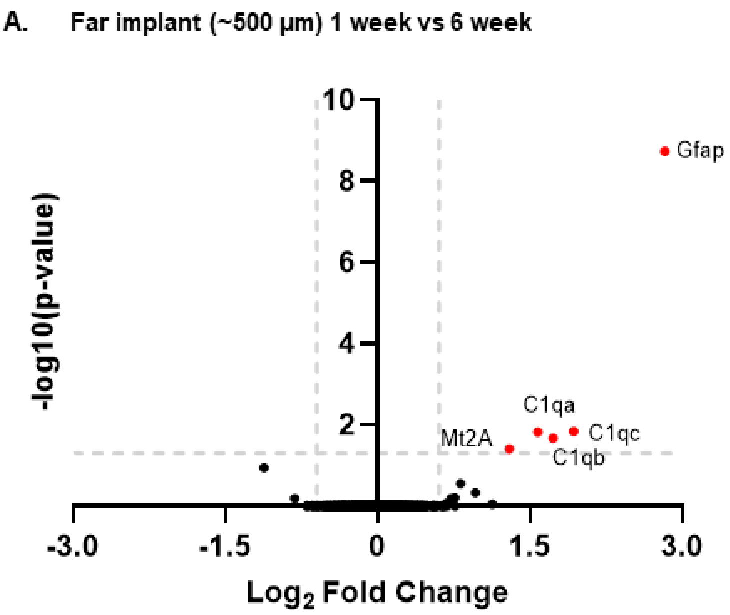

1 week vs 6 week far implant	
Gene Name	Log₂ Fold Change (1 week)
*Gfap****	2.824
*C1qc**	1.927
*C1qb**	1.726
*C1qa**	1.576
*Mt2A**	1.295
Apod	1.129
S100a16	0.963
Rpl39	0.816
Gpx1	0.761
Ttc9b	0.757
Myl6	0.721
Cox6b1	0.683
Rpl37a-ps1	0.682
Tmsb10	0.662
Npc2	0.649
S100a10	0.644
Pcp4	0.632
Ftl1	0.628
Rpl38-ps9	0.589
Cd63	0.573
Nefm	0.563
Anxa3	0.554
Rpl35a	0.552
Cycs	0.549
B2m	0.544
Pdcd5	0.538
Ndufb6	0.534
Fth1	0.533
Scand1	0.486
ENSRNOG00000064200	0.486
Rps25	0.484
Tpt1	0.468
Serf2	0.466
Ndufb5	0.464
Mbp	0.463
Eif1b	0.457
AABR07015346.1	0.456
Rpl17	0.447
Rps27	0.447
Pfdn1	0.444

Supplementary Figure 1. Differentially expressed genes (DEGs) in far regions between 1-week and 6-week implants. **A.** Volcano plot of all DEGs with red spots indicating significant DEGs. Table lists top 40 DEGs ordered by Log_2 Fold Change. Significance p-value < 0.05*, < 0.01**, 0.001***. Total n = 10 rats (n = 7 rats per time point).

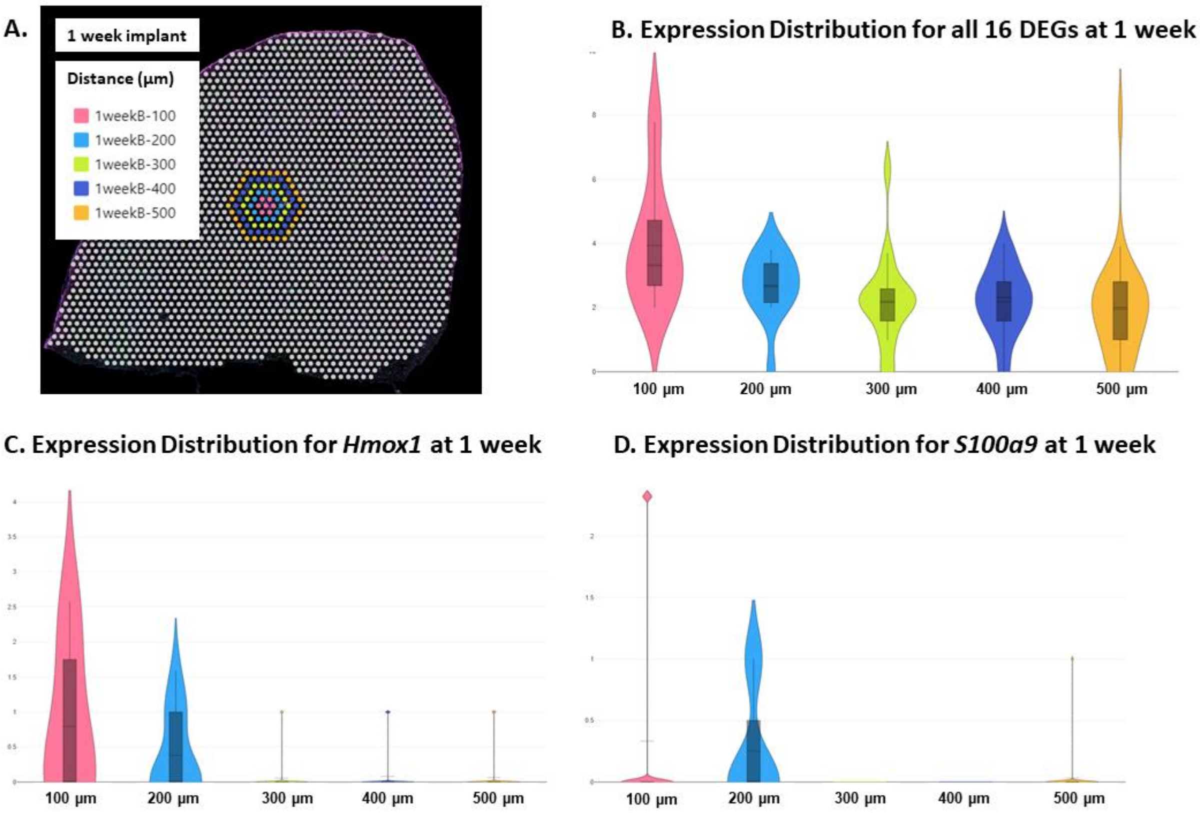

Supplementary Figure 2. Assessment of the expression distribution in a 1-week sample of 16 significant DEGs revealed during differential expression analysis of the near implant regions between 1-week and 6-week tissue sections. **A.** Concentric rings selected from implant site at distances of 100 µm, 200µm, 300µm, 400 µm and 500 µm. **B.** Combined expression distribution of all 16 DEGs. **C.** Isolated expression distribution of Hmox1 indicates most expression of the gene within 100 µm of implant site. **D.** Isolated expression distribution of S100a9 indicates most gene expression at 200 µm ring.

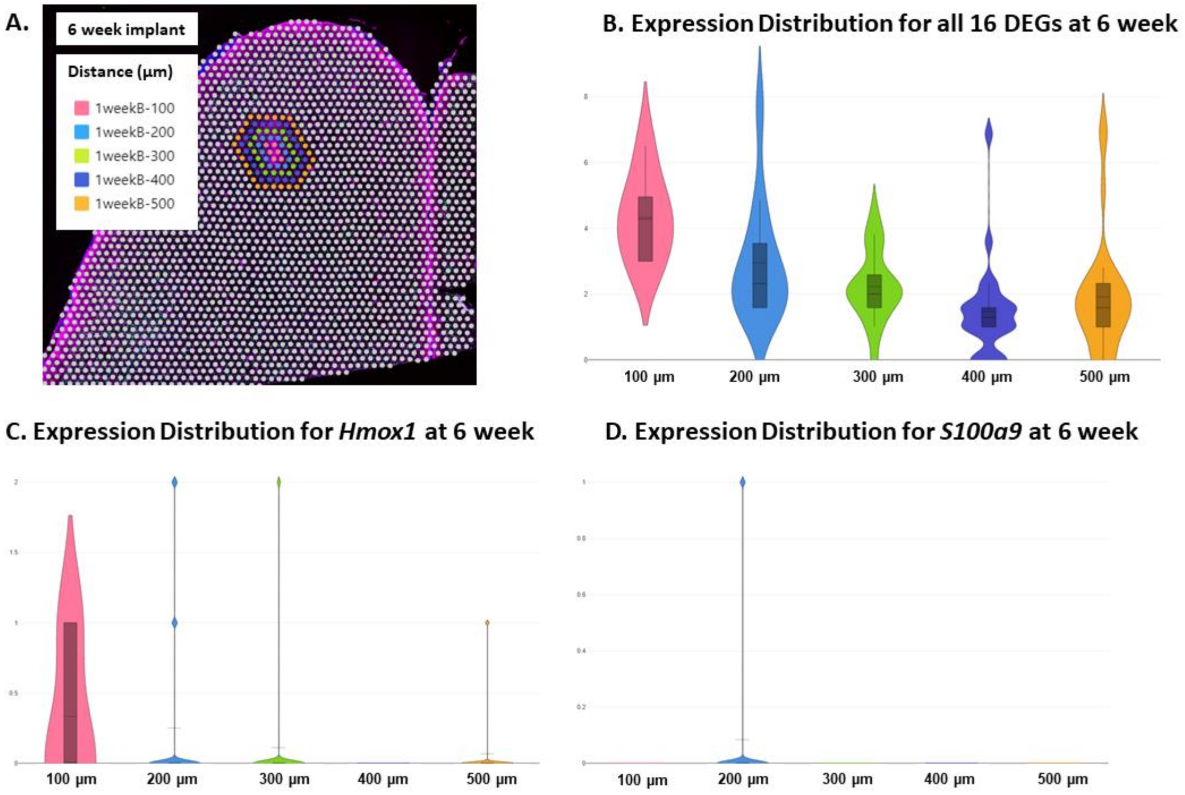

Supplementary Figure 3. Assessment of the expression distribution in a 6-week of 16 significant DEGs revealed during differential expression analysis of the near implant regions between 1-week and 6-week tissue sections. **A.** Concentric rings selected from implant site at distances of 100 µm, 200µm, 300µm, 400 µm and 500 µm. **B.** Combined expression distribution of all 16 DEGs. **C.** Isolated expression distribution of Hmox1 indicates most expression of the gene within 100 µm of implant site. **D.** Isolated expression distribution of S100a9 indicates diminished gene expression at 200 µm ring.

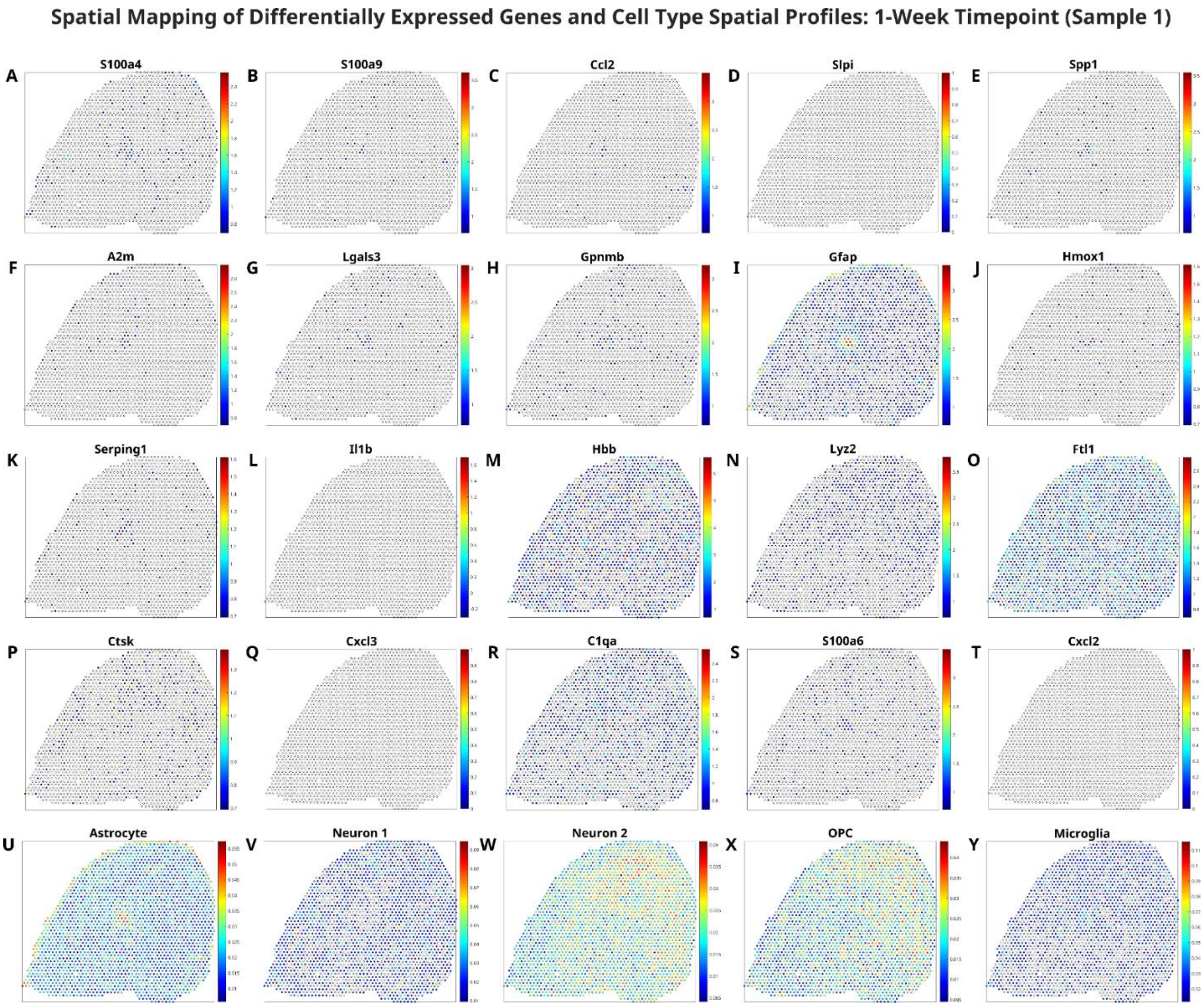

Supplementary Figure 4 (1/14). Spatial distribution of differentially expressed genes and cell type profiles. **(A–T)** Top panels display the spatial expression maps for 20 notable differentially expressed genes across tissue sections. **(U–Y)** Bottom panels show the spatial density and distribution of astrocytes, neurons, OPCs, and microglia, generated using non-negative matrix factorization. This figure is continued on pages 6-18.

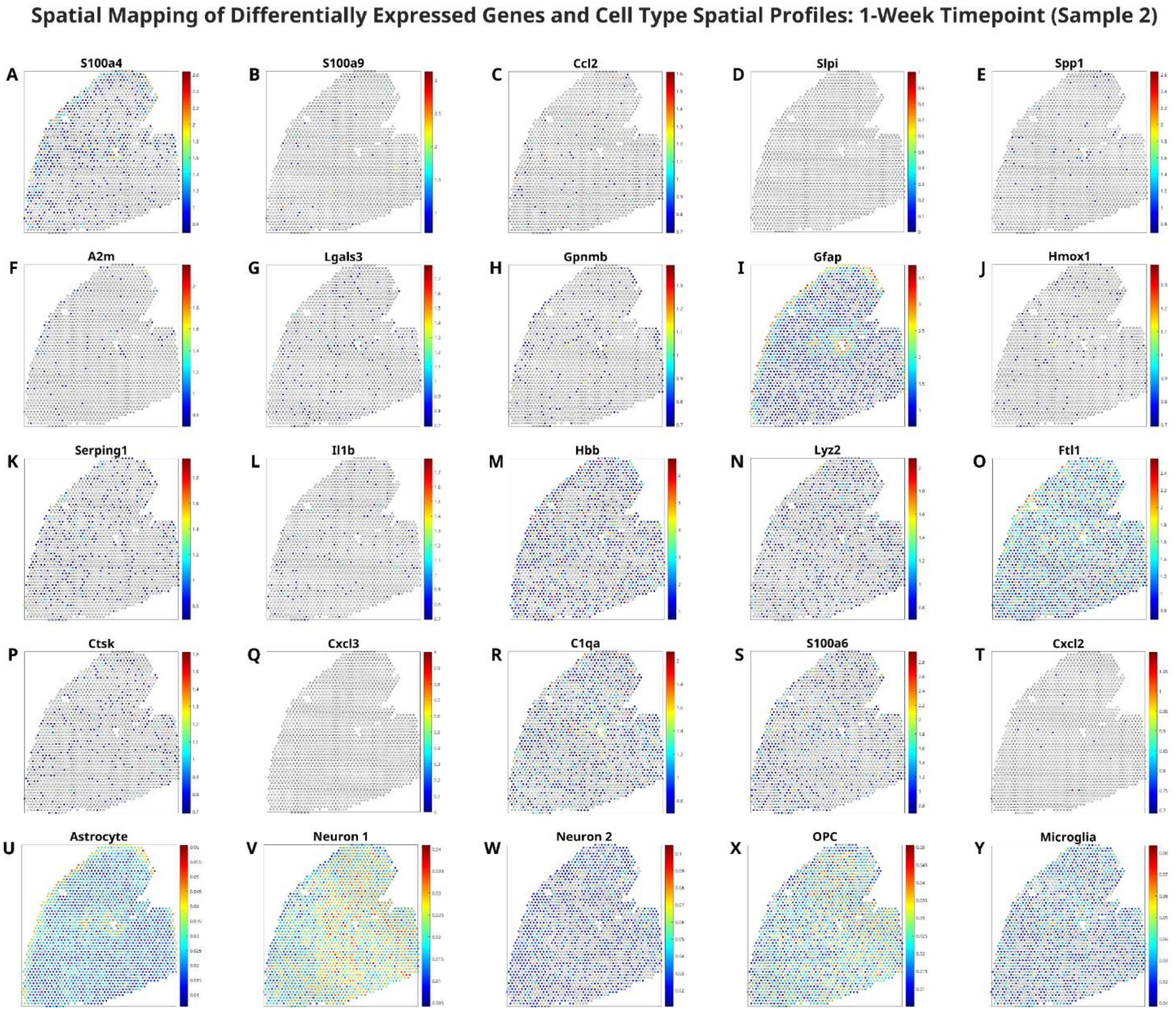

Supplementary Figure 4 (Contd., 2/14). Spatial distribution of differentially expressed genes and cell type profiles. **(A–T)** Top panels display the spatial expression maps for 20 notable differentially expressed genes across tissue sections. **(U–Y)** Bottom panels show the spatial density and distribution of astrocytes, neurons, OPCs, and microglia, generated using non-negative matrix factorization. This figure is continued on pages 6-18.

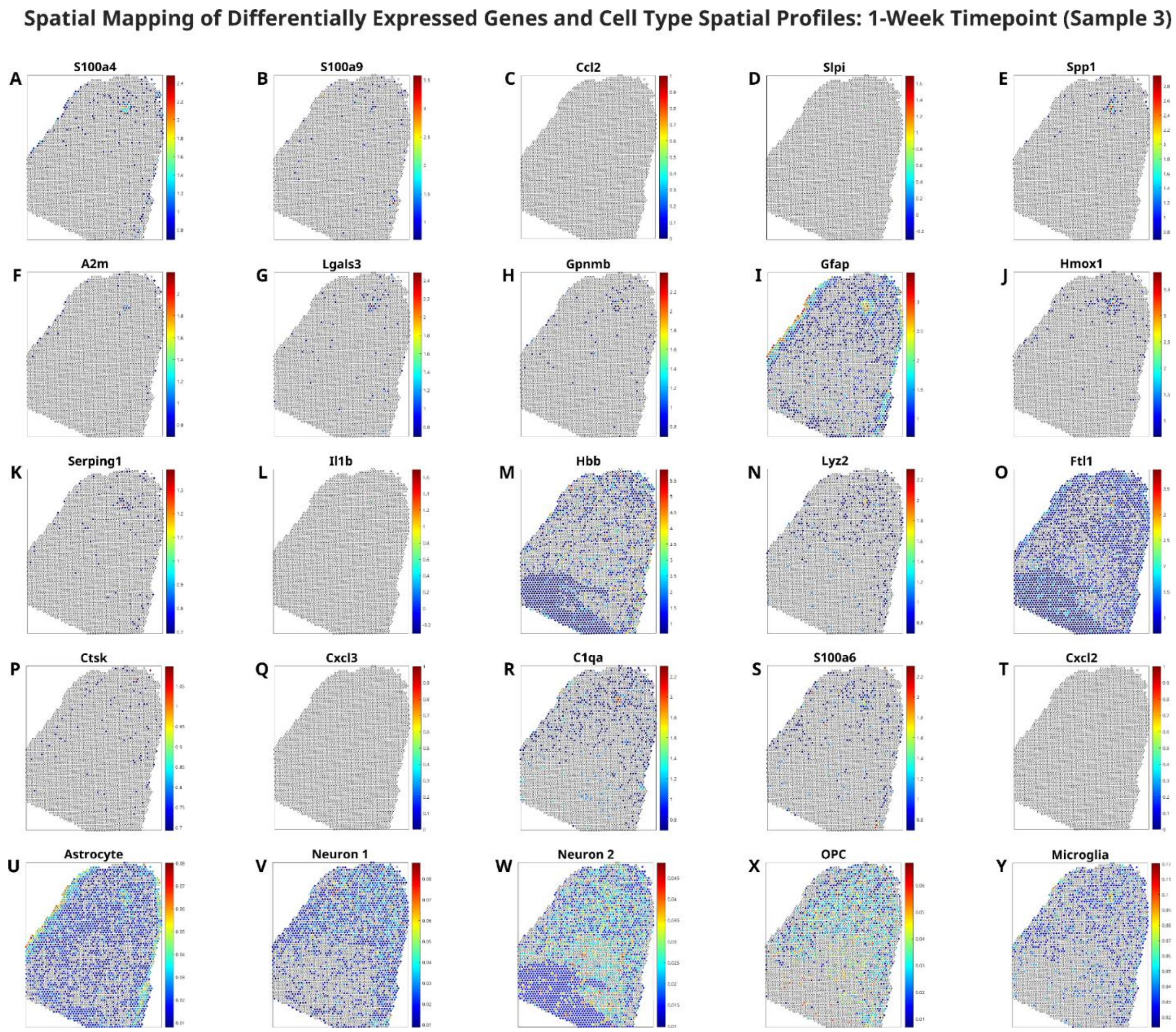

Supplementary Figure 4 (Contd., 3/14). Spatial distribution of differentially expressed genes and cell type profiles. **(A–T)** Top panels display the spatial expression maps for 20 notable differentially expressed genes across tissue sections. **(U–Y)** Bottom panels show the spatial density and distribution of astrocytes, neurons, OPCs, and microglia, generated using non-negative matrix factorization. This figure is continued on pages 6-18.

Spatial Mapping of Differentially Expressed Genes and Cell Type Spatial Profiles: 1-Week Timepoint (Sample 4)

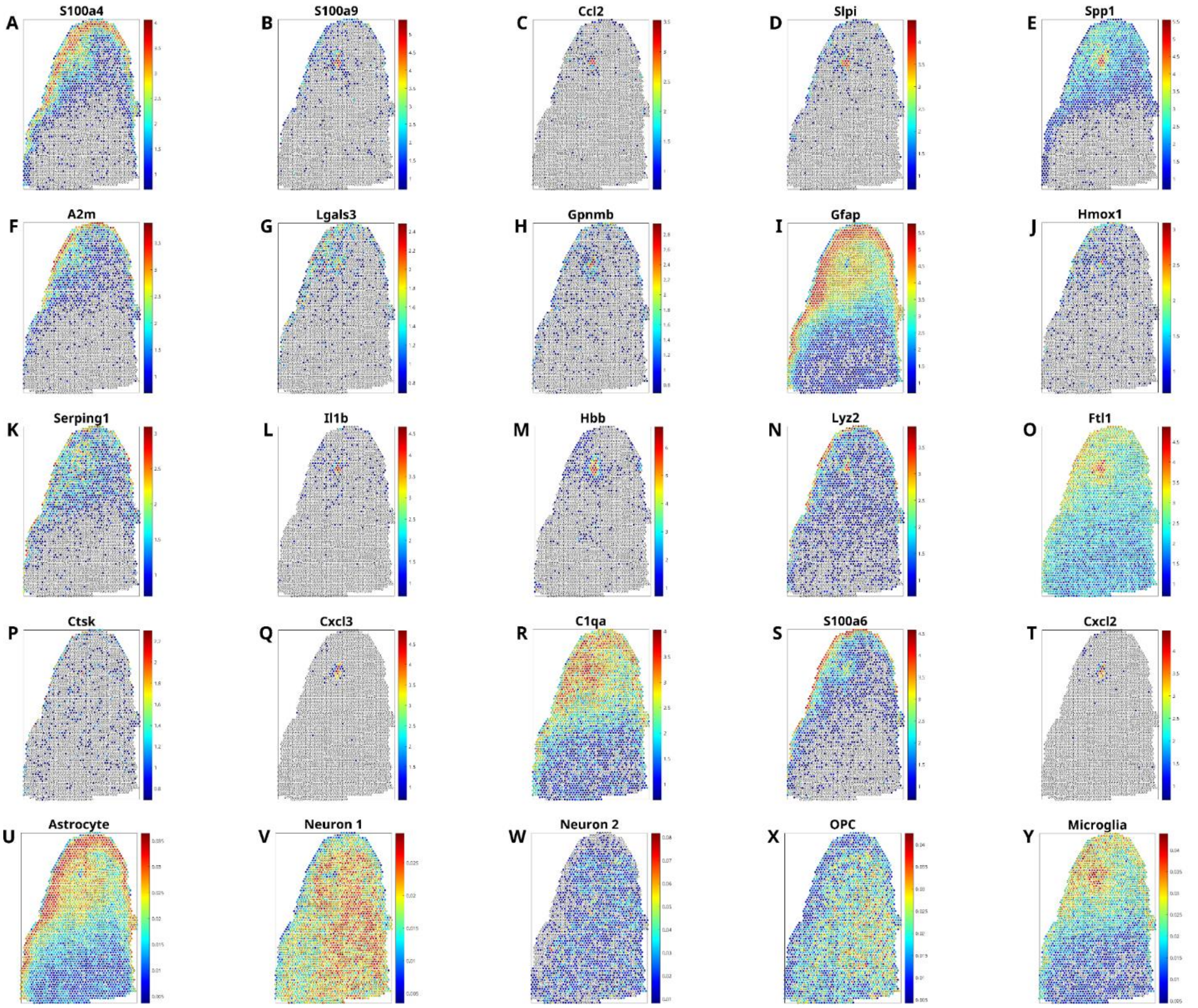

Supplementary Figure 4 (Contd., 4/14). Spatial distribution of differentially expressed genes and cell type profiles. **(A–T)** Top panels display the spatial expression maps for 20 notable differentially expressed genes across tissue sections. **(U–Y)** Bottom panels show the spatial density and distribution of astrocytes, neurons, OPCs, and microglia, generated using non-negative matrix factorization. This figure is continued on pages 6-18.

Spatial Mapping of Differentially Expressed Genes and Cell Type Spatial Profiles: 1-Week Timepoint (Sample 5)

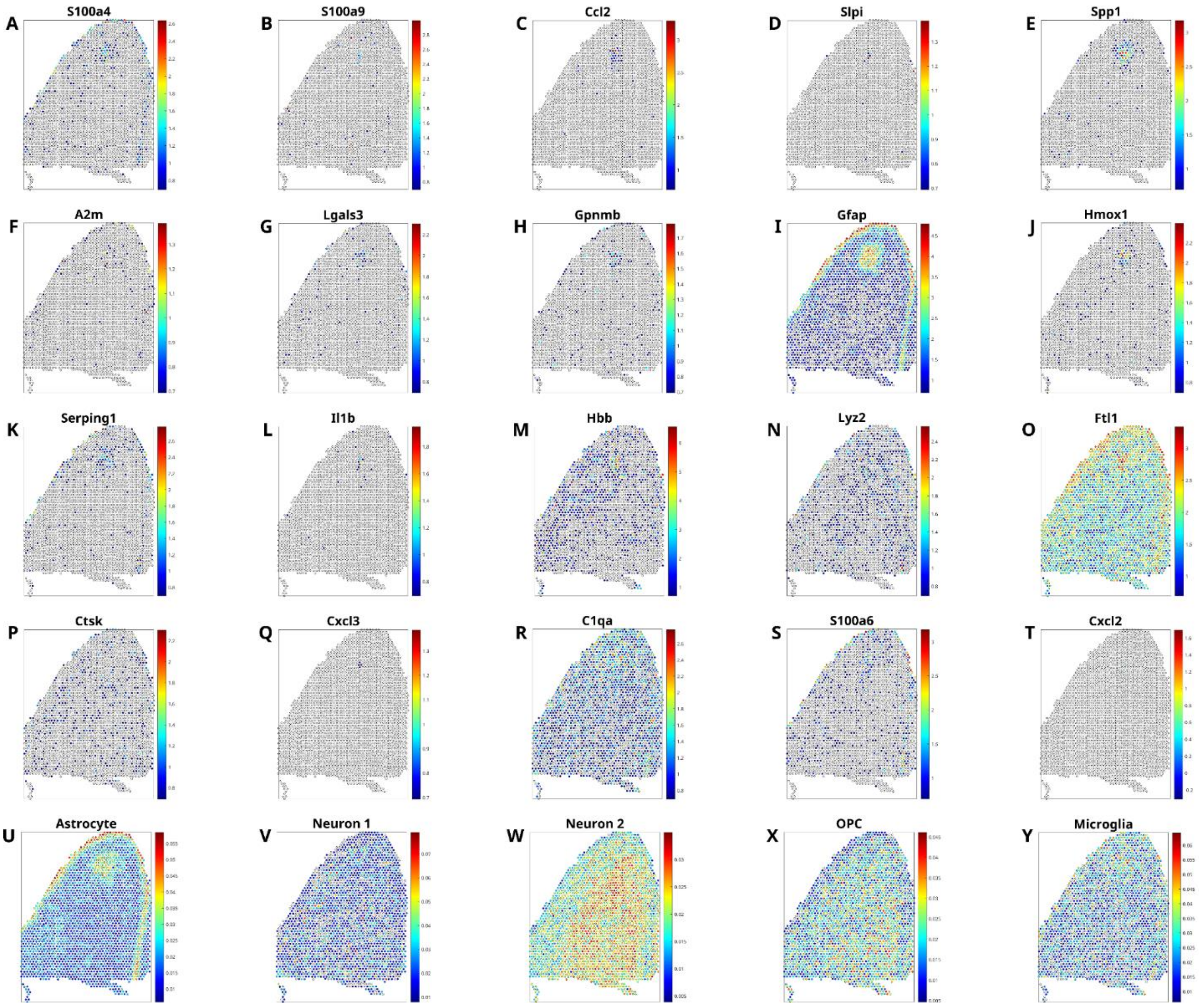

Supplementary Figure 4 (Contd., 5/14). Spatial distribution of differentially expressed genes and cell type profiles. **(A–T)** Top panels display the spatial expression maps for 20 notable differentially expressed genes across tissue sections. **(U–Y)** Bottom panels show the spatial density and distribution of astrocytes, neurons, OPCs, and microglia, generated using non-negative matrix factorization. This figure is continued on pages 6-18.

Spatial Mapping of Differentially Expressed Genes and Cell Type Spatial Profiles: 1-Week Timepoint (Sample 6)

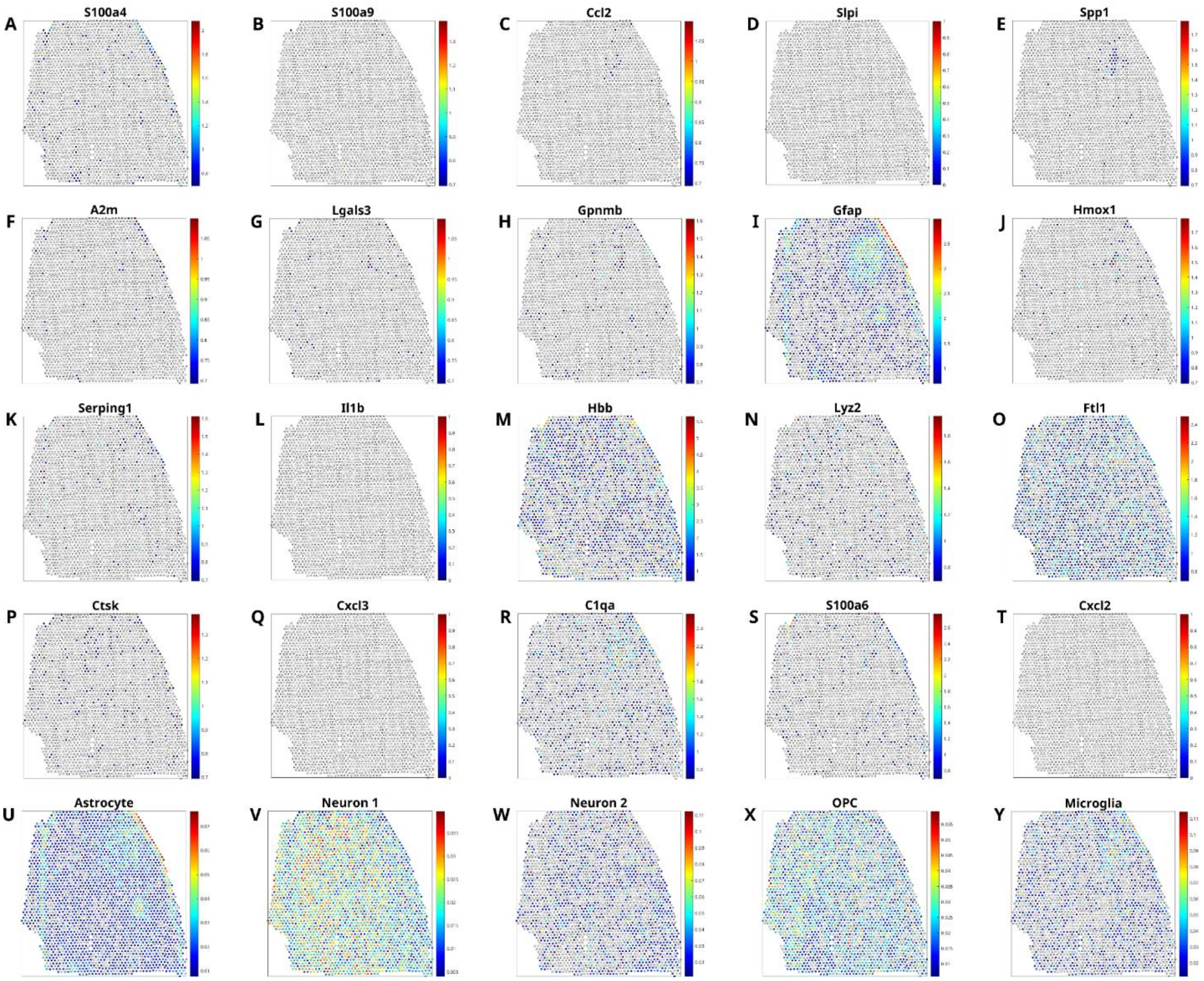

Supplementary Figure 4 (Contd., 6/14). Spatial distribution of differentially expressed genes and cell type profiles. **(A–T)** Top panels display the spatial expression maps for 20 notable differentially expressed genes across tissue sections. **(U–Y)** Bottom panels show the spatial density and distribution of astrocytes, neurons, OPCs, and microglia, generated using non-negative matrix factorization. This figure is continued on pages 6-18.

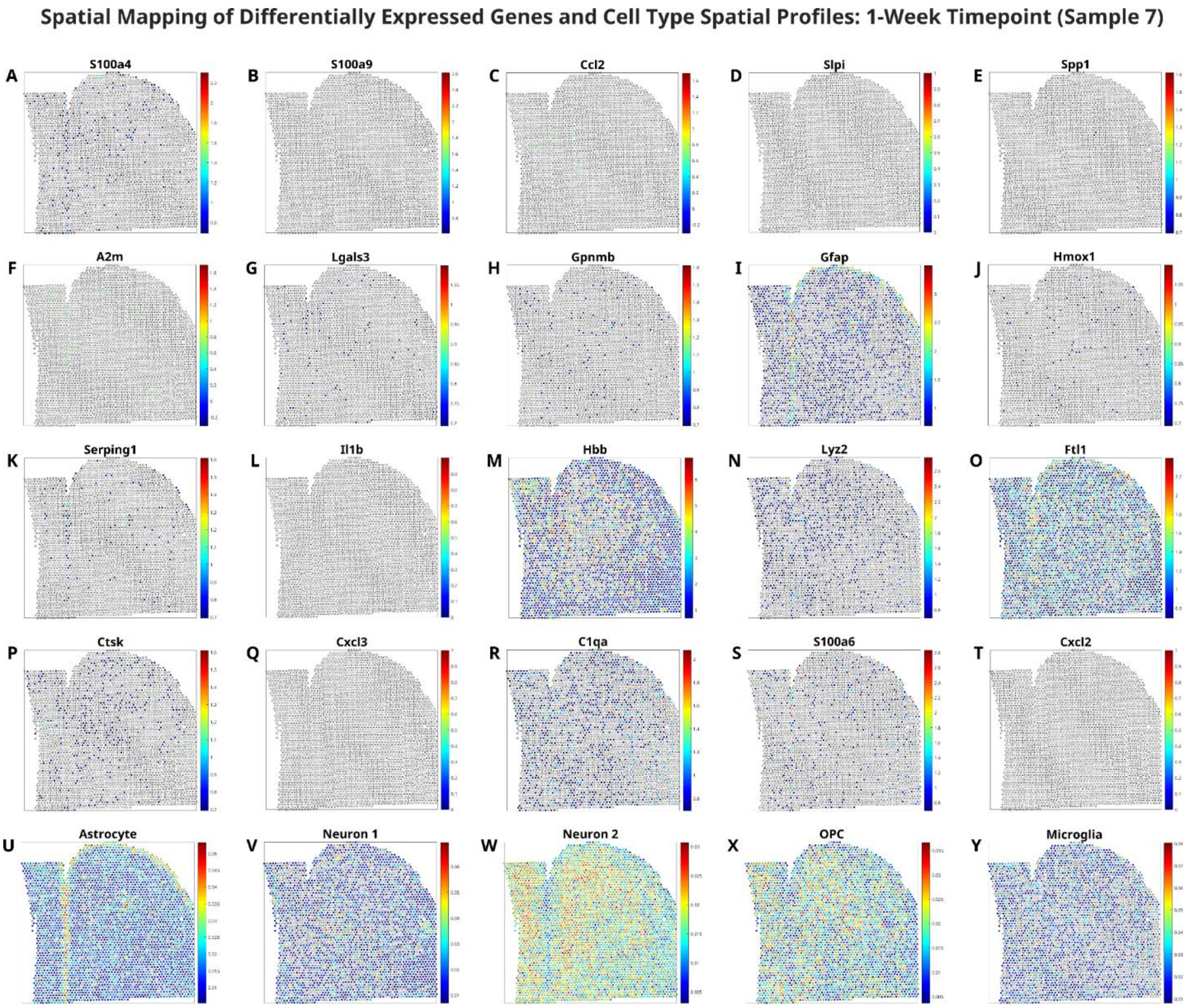

Supplementary Figure 4 (Contd., 7/14). Spatial distribution of differentially expressed genes and cell type profiles. **(A–T)** Top panels display the spatial expression maps for 20 notable differentially expressed genes across tissue sections. **(U–Y)** Bottom panels show the spatial density and distribution of astrocytes, neurons, OPCs, and microglia, generated using non-negative matrix factorization. This figure is continued on pages 6-18.

Spatial Mapping of Differentially Expressed Genes and Cell Type Spatial Profiles: 6-Week Timepoint (Sample 8)

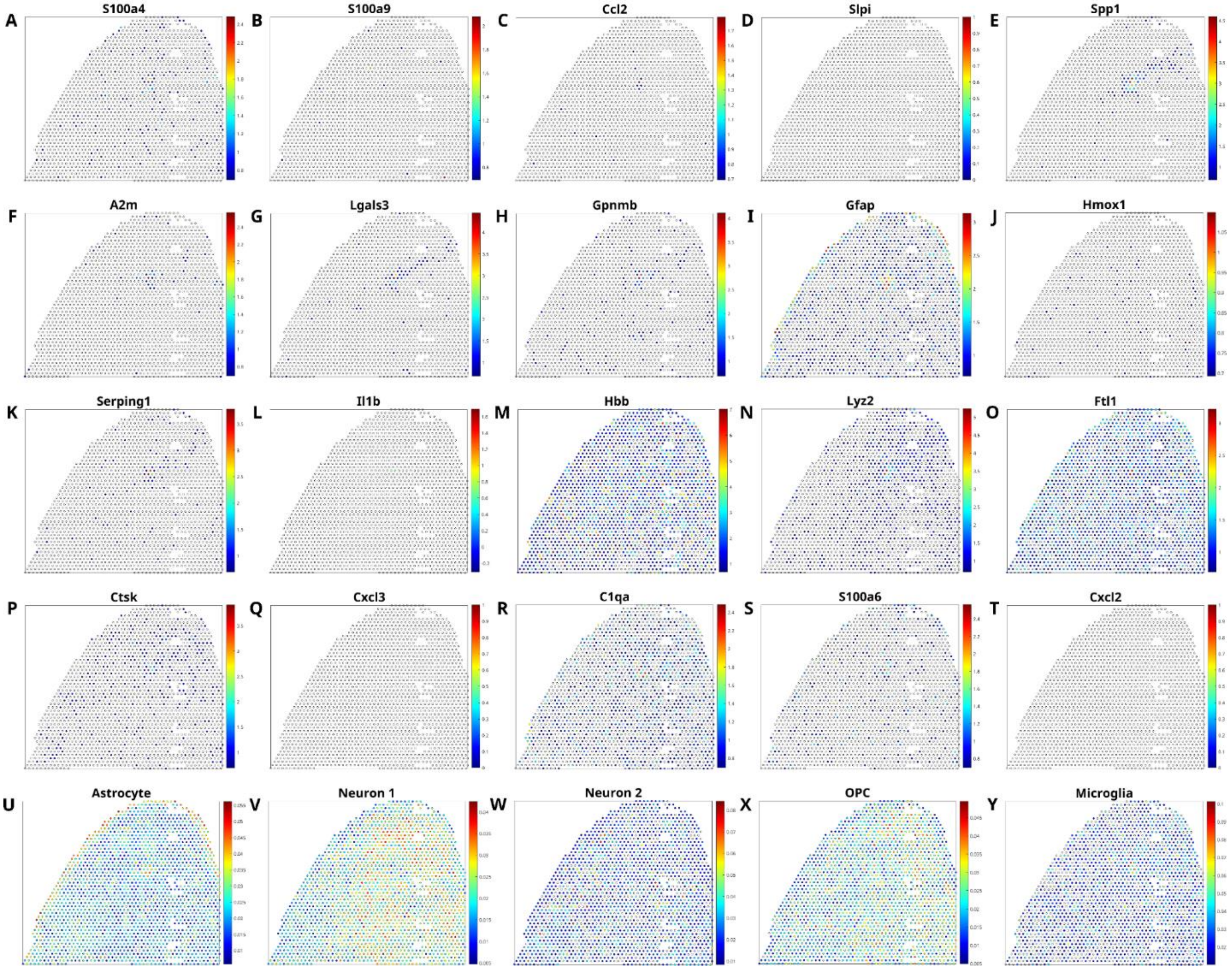

Supplementary Figure 4 (Contd., 8/14). Spatial distribution of differentially expressed genes and cell type profiles. **(A–T)** Top panels display the spatial expression maps for 20 notable differentially expressed genes across tissue sections. **(U–Y)** Bottom panels show the spatial density and distribution of astrocytes, neurons, OPCs, and microglia, generated using non-negative matrix factorization. This figure is continued on pages 6-18.

Spatial Mapping of Differentially Expressed Genes and Cell Type Spatial Profiles: 6-Week Timepoint (Sample 9)

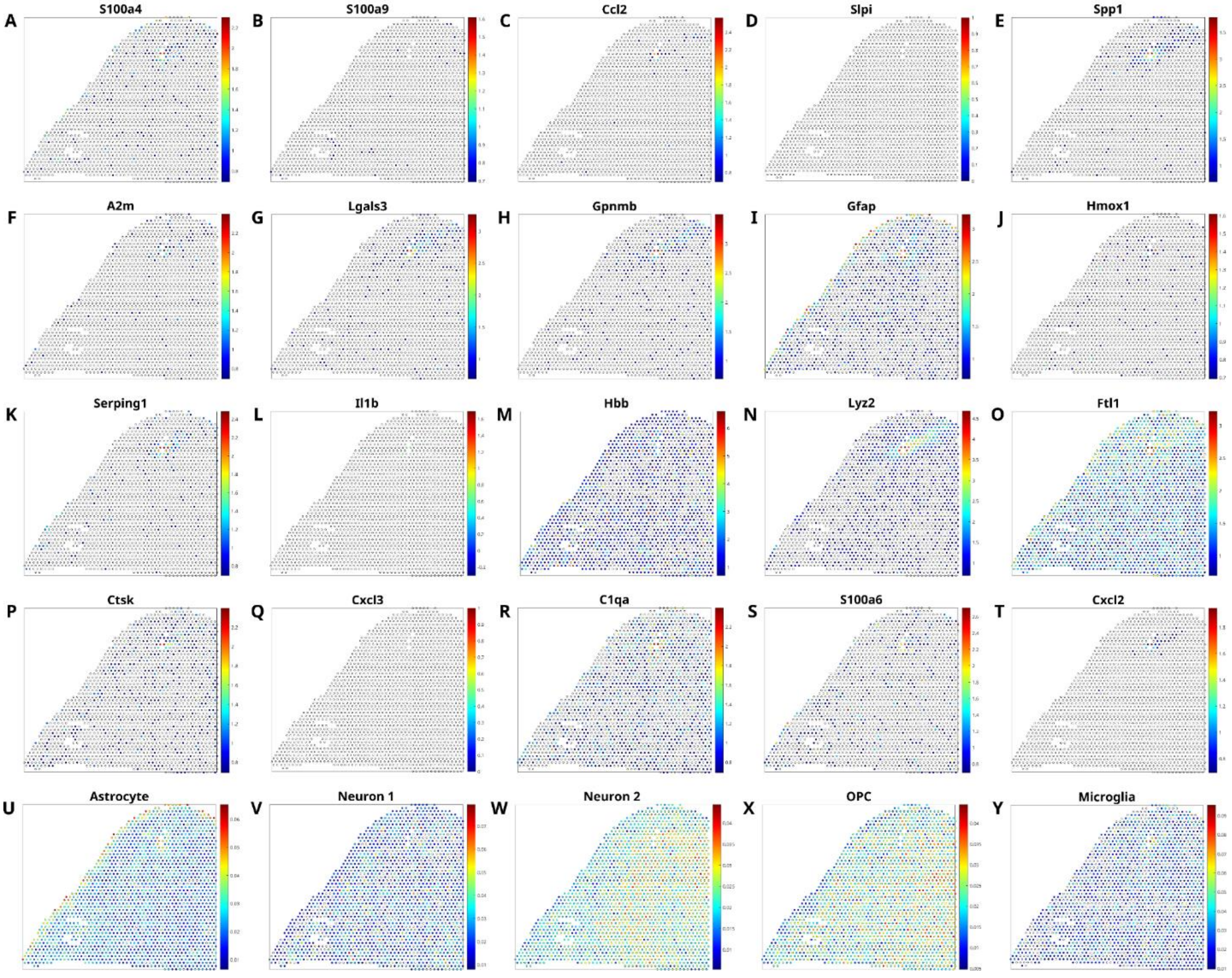

Supplementary Figure 4 (Contd., 9/14). Spatial distribution of differentially expressed genes and cell type profiles. **(A–T)** Top panels display the spatial expression maps for 20 notable differentially expressed genes across tissue sections. **(U–Y)** Bottom panels show the spatial density and distribution of astrocytes, neurons, OPCs, and microglia, generated using non-negative matrix factorization. This figure is continued on pages 6-18.

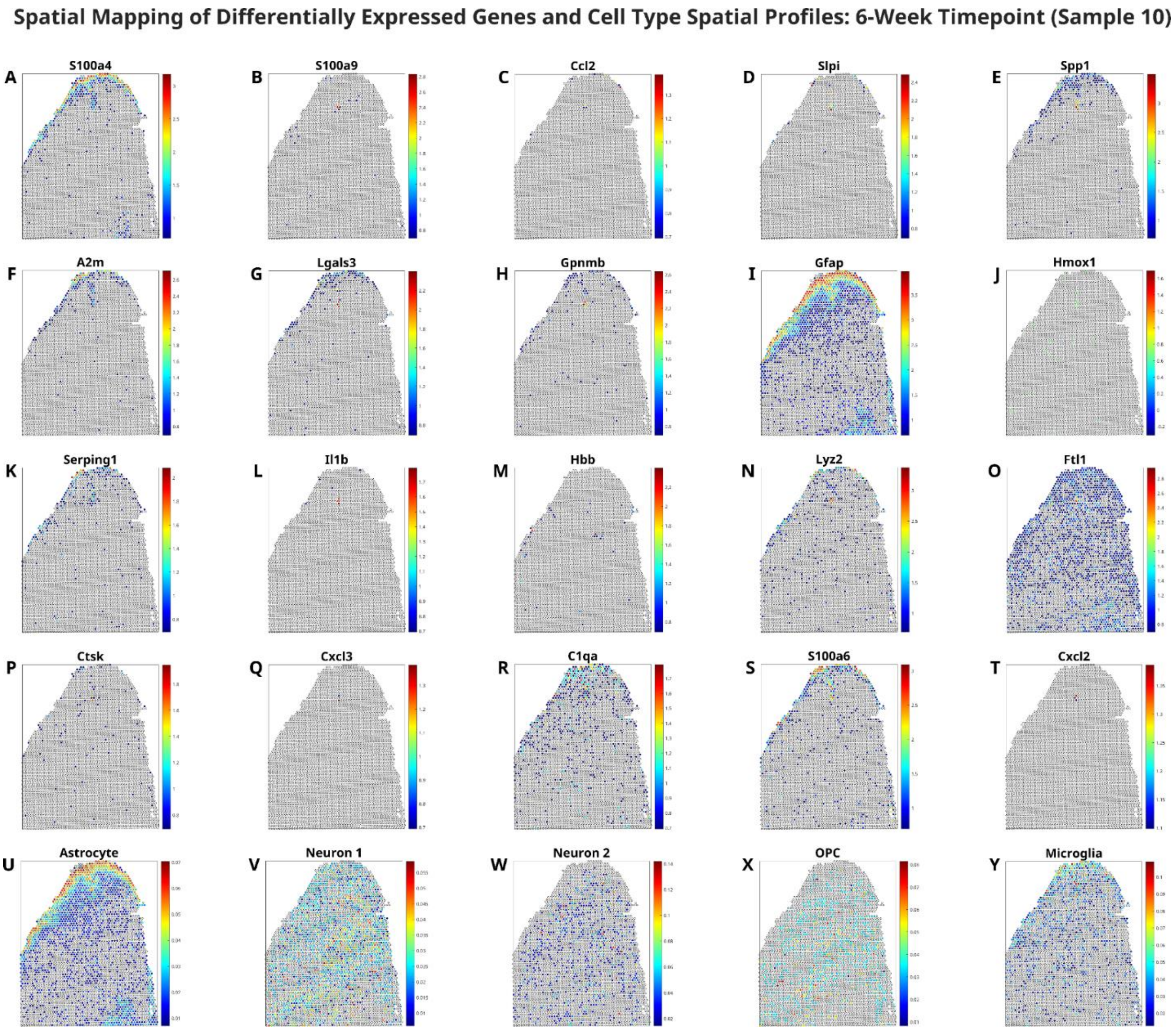

Supplementary Figure 4 (Contd., 10/14). Spatial distribution of differentially expressed genes and cell type profiles. **(A–T)** Top panels display the spatial expression maps for 20 notable differentially expressed genes across tissue sections. **(U–Y)** Bottom panels show the spatial density and distribution of astrocytes, neurons, OPCs, and microglia, generated using non-negative matrix factorization. This figure is continued on pages 6-18.

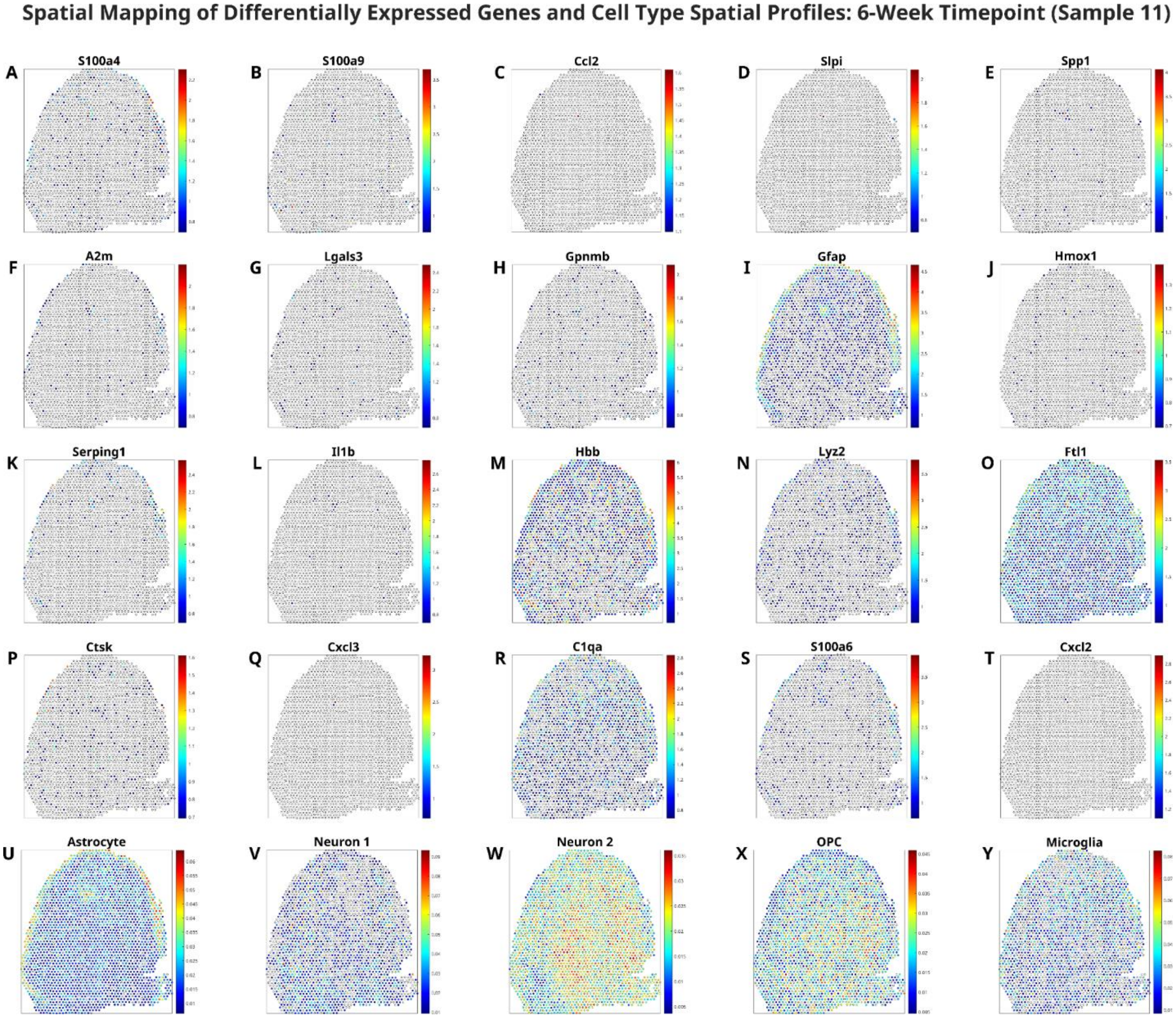

Supplementary Figure 4 (Contd., 11/14). Spatial distribution of differentially expressed genes and cell type profiles. **(A–T)** Top panels display the spatial expression maps for 20 notable differentially expressed genes across tissue sections. **(U–Y)** Bottom panels show the spatial density and distribution of astrocytes, neurons, OPCs, and microglia, generated using non-negative matrix factorization. This figure is continued on pages 6-18.

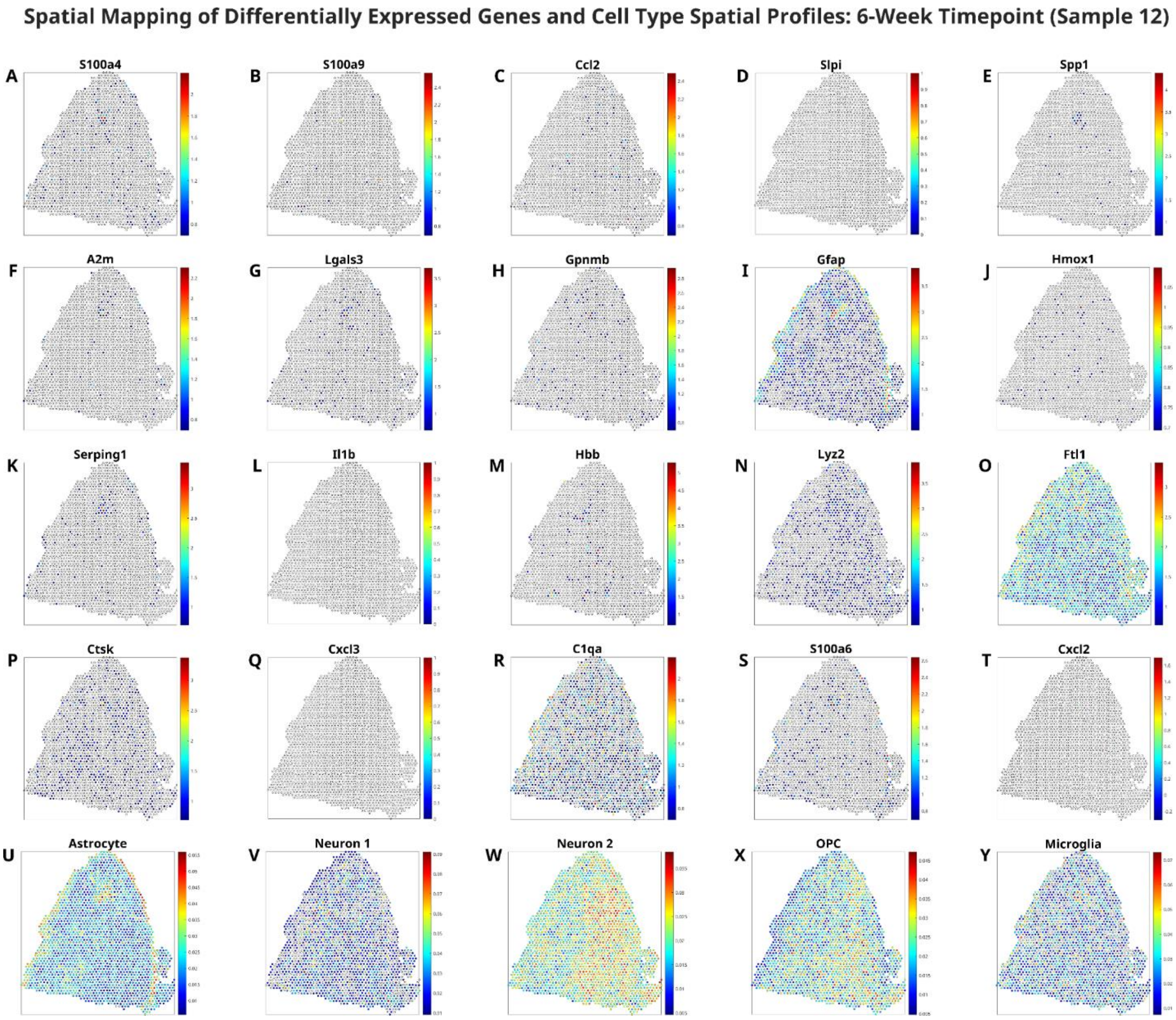

Supplementary Figure 4 (Contd., 12/14). Spatial distribution of differentially expressed genes and cell type profiles. **(A–T)** Top panels display the spatial expression maps for 20 notable differentially expressed genes across tissue sections. **(U–Y)** Bottom panels show the spatial density and distribution of astrocytes, neurons, OPCs, and microglia, generated using non-negative matrix factorization. This figure is continued on pages 6-18.

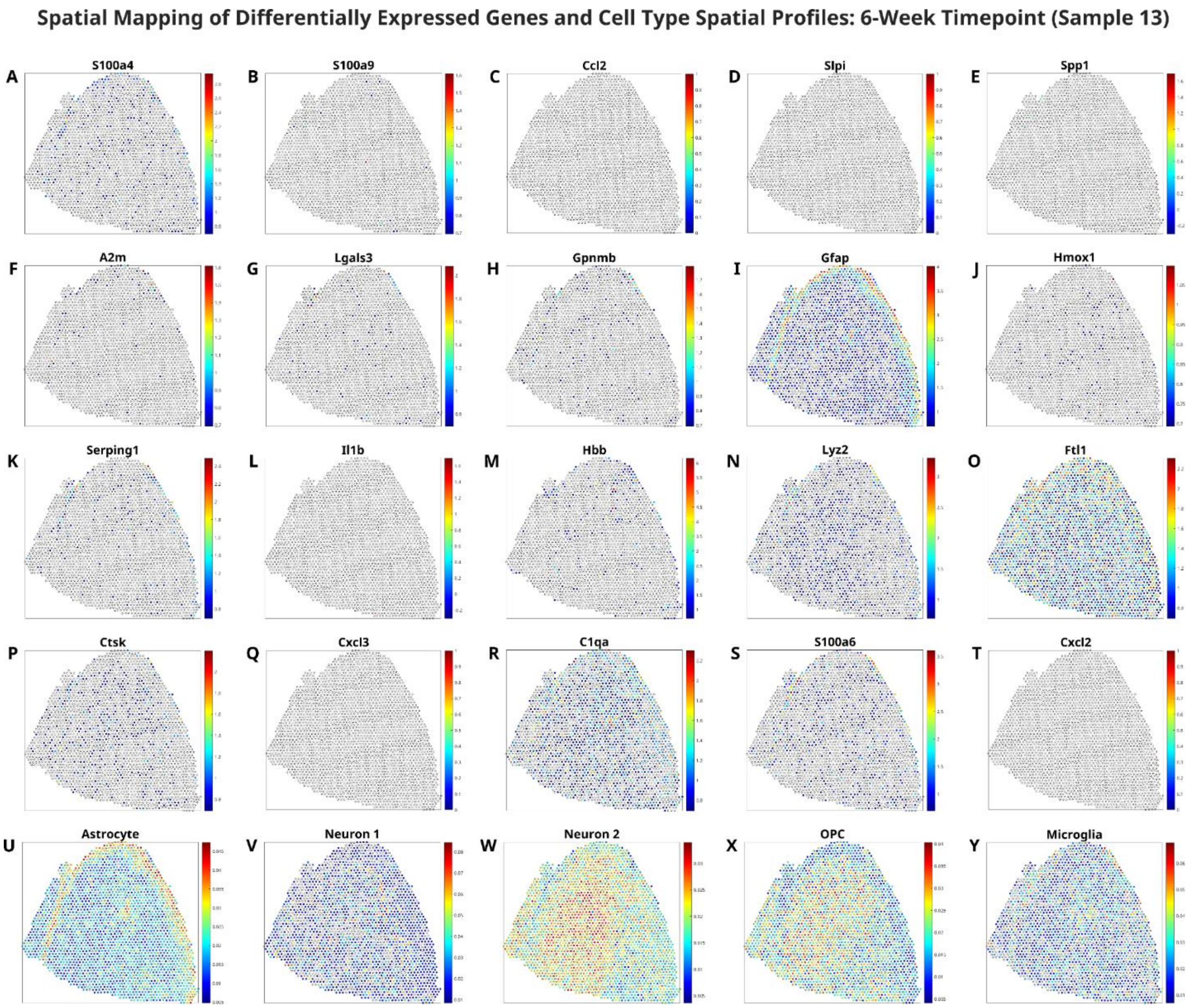

Supplementary Figure 4 (Contd., 13/14). Spatial distribution of differentially expressed genes and cell type profiles. **(A–T)** Top panels display the spatial expression maps for 20 notable differentially expressed genes across tissue sections. **(U–Y)** Bottom panels show the spatial density and distribution of astrocytes, neurons, OPCs, and microglia, generated using non-negative matrix factorization. This figure is continued on pages 6-18.

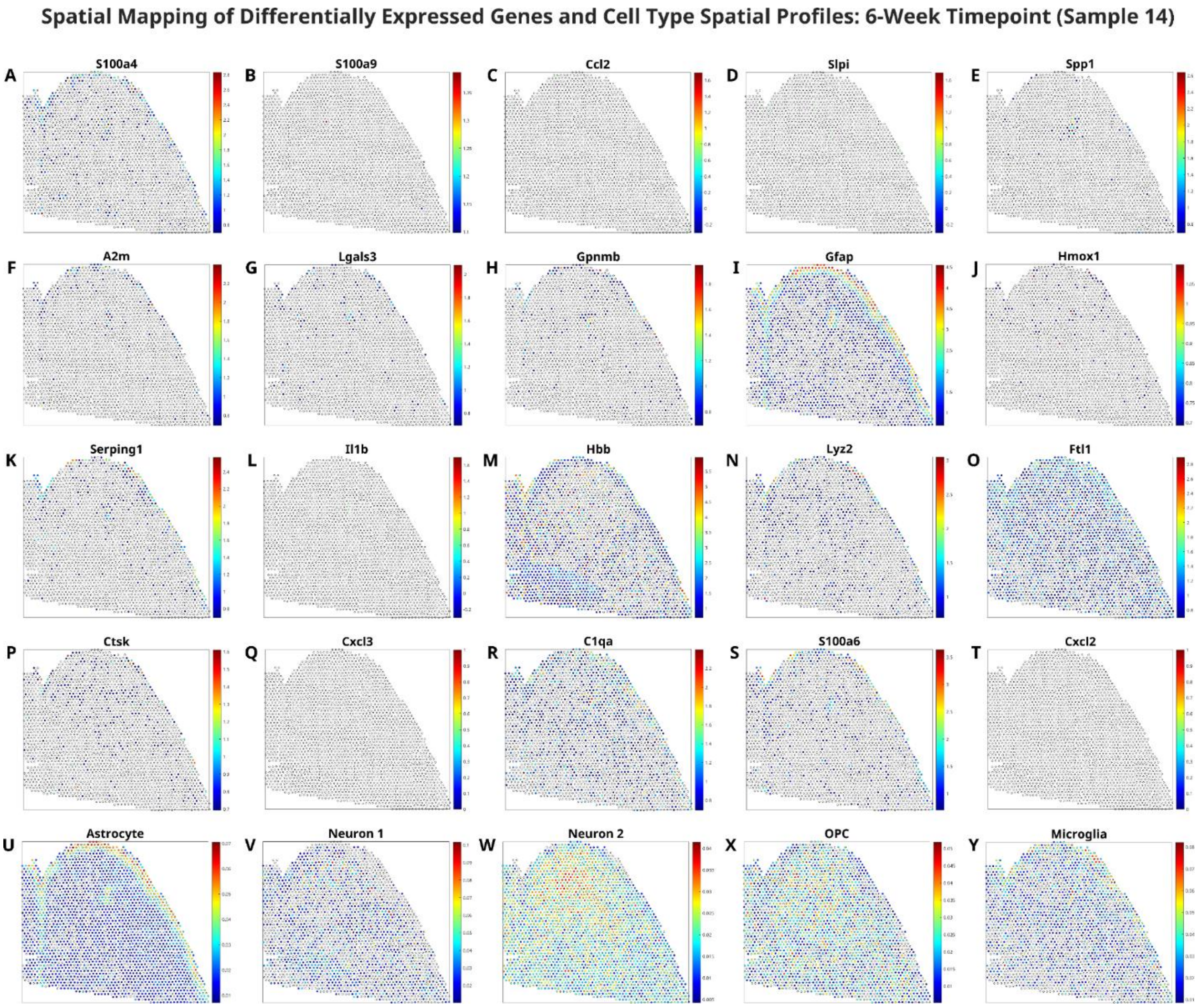

Supplementary Figure 4 (Contd., 14/14). Spatial distribution of differentially expressed genes and cell type profiles. **(A–T)** Top panels display the spatial expression maps for 20 notable differentially expressed genes across tissue sections. **(U–Y)** Bottom panels show the spatial density and distribution of astrocytes, neurons, OPCs, and microglia, generated using non-negative matrix factorization. This figure is continued on pages 6-18.
