# Supplementary material for: Spatial transcriptomics on an expanded dataset at the brain-electrode interface: exploration of variability and identification of novel biomarkers
Source: Front Neurosci. 2026 Jun 15;20:1852774. doi: 10.3389/fnins.2026.1852774 (PMC13311015; doi:10.3389/fnins.2026.1852774)
Supplement: Supplementary file 3 [file Data_Sheet_3.pdf]

Batch Correction in Spatial Transcriptomics Data: Overview, Comparison and Results

1. Summary

Batch effects are pervasive in high-dimensional transcriptomic data like that obtained by the 10x Genomics Visium platform. These effects can arise from various sources including operator or experimenter handling (operator), slide-to-slide variability (Slide ID), processing or sequencing run differences and spatial artifacts (tissue thickness, RNA diffusion, imaging inconsistencies). Spatial transcriptomics data often contain a mixture of technical, biological, and spatially-driven batch effects. It is important to inspect data for obvious batch effects to avoid misinterpreting results and obscuring biological results.

In this document, we assess three batch correction pipelines: (1) Seurat integration, (2) Harmony and (3) Limma (Linear Models for Microarray Data), for our Visium (10x Genomics) data. We also aggregated and loaded the data in Loupe Browser (10x Genomics) for visualization and comparison. Batch effects modelling was done for as many of the reported variables, including operator, slide IDs, and processing dates. Our overall goal was to develop an efficient method to obtain suitable gene expression data, with minimal batch effects, as input for downstream gene expression analyses such as differential gene expression analysis. As mentioned in the main manuscript, both Seurat and Harmony pipelines produced integrated data outputs that were not suitable for downstream differential expression analysis, and better fitted for clustering and visualization. Meanwhile, Limma produced an output usable for downstream differential expression, but due to its linear model framework (assuming linear distribution of RNA-seq data), this output would require additional testing, transformations and corrections prior to use in differential expression analysis for accurate results. We inspected the UMAPs of our aggregated Visium dataset in LoupeBrowser and found that although an operator-based batch effect was noticeable, other variables had minor effects on the overall data. Additionally, the number of spots and regions of interest selected for differential expression contained a small number of “operator”-effected spots. The Loupe Browser software also performs correction and normalization of the data in selected comparisons prior to differential expression analysis.

Considering the broader limitations of the three batch-correction pipelines—Seurat integration, Harmony, and Limma—and weighing the greater efficiency, ease and accuracy of Loupe Browser, we proceeded with data aggregation and Loupe Browser facilitated differential expression analysis of our spatial transcriptomics data. Although the data in Loupe Browser showed apparent clustering for operator, and minimal clustering for other variables, the spots generated from “Operator 1”-affected samples for downstream DE analysis was a relatively low number (~78 spots) compared to all the mapped spots (~32,900 spots) plotted on the UMAP to visualize clustering. Furthermore, Loupe Browser utilizes additional correction and normalization of the loaded dataset in the differential expression analysis pipeline. Overall, the scope of this study allowed us to explore batch-correction pipelines for high-dimensional transcriptomic data and

choose an efficient and low-risk method to obtain gene expression data for downstream differential expression analysis. However, future studies could further extend our findings by testing, correcting and transforming the Limma output for use in differential expression pipelines with this dataset or other similar high-dimensional transcriptomic data.

2. Dataset and variables

The following variables and details were considered during batch correction of our spatial transcriptomics data:

- Sample: 14 rat tissue sections (each from a distinct implanted rat brain)
- Time Point: n = 7 samples of 1-week implants and n = 7 samples of 6-week implants
- Operator: The 14 samples were collected by 2 experimenters or operators: operator 1 collected 4 samples (two 1-week and two 6-week implant samples reported previously(1)), and operator 2 collected an additional 10 samples (five 1-week and five 6-week implant samples).
- Slide ID/processing date: Each Visium slide has 4 capture sites, containing up to 4 different samples. Each Visium slide has a unique serial number. A total of n = 5 slides were used to generate the data reported here. Each slide ID was processed on an individual processing date. Operator 1 collected the data in 2020 and 2021 whereas operator 2 collected data across 2024-2025.

3. Methods, Results and Discussion

Batch effects modelling was done for as many of the reported variables, including samples, operators, and slide IDs, while preserving the variation between time points. Three main pipelines of batch correction were employed: Seurat Integration, Harmony and Limma (Linear Models for Microarray Data). Lastly, we plotted and compared UMAPs generated from the LoupeBrowser (10x Genomics) platform.

3.2. Seurat Integration

Seurat's integration pipeline aligns multiple datasets using 'anchors' — pairs of cells that are mutual nearest neighbors across datasets(2). It corrects expression values within an 'integrated' assay, enabling unified clustering and visualization. Seurat's integration framework was developed to address this problem by aligning datasets into a shared space, such that cells or spots with similar transcriptional profiles group together, regardless of their batch or sample origin.

3.2.1. Workflow

A. Preprocessing each dataset:
   - For each dataset (`Seurat` object), normalization and feature selection are performed independently.
   - Usually, `SCTransform()` or `NormalizeData()` + `FindVariableFeatures()` are used.
   - This ensures each dataset is internally normalized before alignment.

B. Finding integration Features:

- The function `SelectIntegrationFeatures()` identifies genes that are informative across datasets.
- These are typically highly variable genes that are consistently detected across samples.

C. Finding Integration anchors:

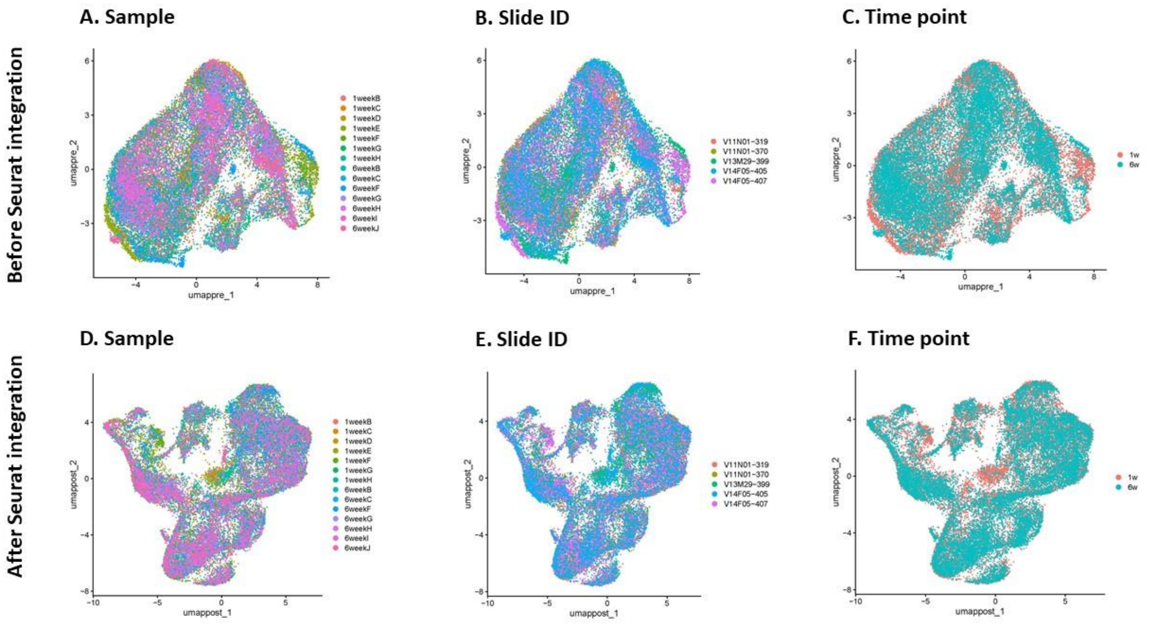

Figure 1. Seurat integration UMAP plots. A, B, C. Pre-integration UMAPs: Spots from each sample are widespread regardless of the timepoint the sample belongs to. D, E, F. Post integration UMAPs: The time points are most prominently more aligned, with the spots from 1-week samples (Orange to green) clustering together, showing a successful integration.

- The key step of integration: Seurat identifies "anchors" between datasets using the `FindIntegrationAnchors()` function.
- Anchors are pairs of cells (or spots) from different datasets that represent the same biological state or type, found using mutual nearest neighbors (MNN) in the PCA space of each dataset.
- This step relies on Canonical Correlation Analysis (CCA) or Reciprocal PCA (RPCA) to project data into comparable subspaces.

D. Data Integration:

- Once anchors are identified, `IntegrateData()` uses them to correct expression values so that shared cell types align across datasets.
- The resulting "integrated assay" represents a corrected low-dimensional representation of the data, with batch effects minimized.

E. Dimensional reduction and Clustering:

- Downstream steps (PCA, UMAP/t-SNE, clustering, differential expression) are then performed on the integrated assay, which now represents shared biological structure rather than batch-specific variation.

The results in Figure 1 show the pre-integration and post-integration results. Each point on the UMAP represents a spot from the representative sample. In the pre-integration UMAPs, the spots are widespread regardless of which timepoint they belong to. In the post-integration UMAP, there is an alignment of spots based on the time point they belong to, spread across the entire cluster structure.

3.2.2 Seurat integration output is not suitable for downstream gene expression analysis after batch correction

A critical limitation of Seurat's integration pipeline is that the integrated assay does not contain raw or normalized gene expression values**.** Instead, the output contains corrected feature representations derived from transformations applied during the integration process. Thus, the output integrated data is better suited for clustering and visualization, not expression quantification. In order to perform differential expression, the Seurat authors recommend using the original raw RNA data which would essentially be the "uncorrected" data (3,4). Further details on these limitations are listed below:

- Integration works in latent space: The integrated data represents corrected latent vectors, not actual mRNA counts. These values are generated through linear combinations of expression patterns guided by anchor pairs and weighted transformations.

- Anchors and corrections are context-dependent: The integrated expression of a gene in one cell depends not just on its original expression but also on how similar cells across batches were aligned. Therefore, it's a non-linear, dataset-dependent transformation.

- Integrated assay is not the batch corrected gene expression matrix: While the "integrated" slot resembles a matrix of expression values, it's a representation optimized for clustering, visualization, and joint analysis — not for differential expression or co-expression. Seurat's authors explicitly recommend using the original ("RNA") assays for DE analysis within or across clusters, not the integrated assay.

- Loss of absolute expression scale: Integration rescales features so that expression values across datasets are comparable in a relative sense. However, the absolute magnitude of gene expression is no longer meaningful — it's been adjusted to equalize distributions across datasets.

3.3. Harmony

Harmony was introduced as a fast, scalable, and flexible algorithm for integrating single-cell datasets while preserving genuine biological variability (5). Unlike Seurat's anchor-based approach, Harmony operates directly in the reduced PCA space rather than manipulating raw or normalized gene expression matrices. It is widely used for visualization and clustering, aligning datasets across batches while retaining biological structure.

3.3.1 Workflow

A. Pre-Processing
   - Normalize and scale your data using standard methods (`NormalizeData`, `ScaleData`, `RunPCA` in Seurat).
   - Perform PCA to obtain cell embeddings that capture dominant sources of variation.

B. Run Harmony
   - Provide the PCA embeddings along with a metadata variable indicating batch identity (e.g., `slide_id`, `processing_date`, or `donor_id`).
   - Harmony iteratively corrects the PCA embeddings to remove batch-specific structure. This includes soft clustering in latent space, followed by batch correction adjustments based on how each batch deviates from the centroid of the cluster.
   - These 2 steps are repeated iteratively until convergence is achieved which means the batch specific differences are minimized in the latent space while preserving the cluster structure.

C. Dimensionality Reduction and clustering:
   - The embeddings can then be used for UMAP/TSNE visualization and clustering.

In **Figure 2**, prior to Harmony correction, clustering seems to be prominently Slide ID dependent and not due to biological similarity between spots from different samples. In the post harmony UMAPs (Figure 2C, D.), the spots have acquired a similar overall structure and do not cluster by slide IDs, but they do still cluster by time points, overlaid on top of each other across different time points. This follows that batch correction puts biologically similar spots closer together, and there will be spots across different time points that have similar gene expression away from the electrode site. The spots occupying the boundaries of the structure might represent the genes that are not as prominent in 6-week implants, and mostly relevant to 1-week implant. Whereas, spots in the center (even though a 2D plot) may encompass the genes most affected by implants across timepoints as these 1-week implant spots are overlaid by 6-week implant spots. Further analysis would be necessary to confirm this.

3.3.2. Harmony output is not suitable for downstream gene expression analysis after batch correction

Similar to Seurat integration, the data output from Harmony is better suited for clustering and visualization instead of actual expression quantification. Harmony does not operate in expression space, and instead, it modifies low-dimensional representations (PCA coordinates). Therefore, it does not compute nor store a "batch-corrected" expression matrix. Further details are listed below:

- No Mapping Backward: PCA embeddings are a linear projection of gene expression data (`XW` where `W` is the PCA rotation matrix). Harmony alters these embeddings in a way that does not correspond to a valid linear transformation of the original gene expression matrix. Thus, there's no inverse transformation to recover corrected counts.

- Local Shifts Are Not Gene-Specific**:** Corrections occur in the latent space, not per gene. The adjustments are cluster- and batch-specific translations, not per-gene scaling or subtraction.

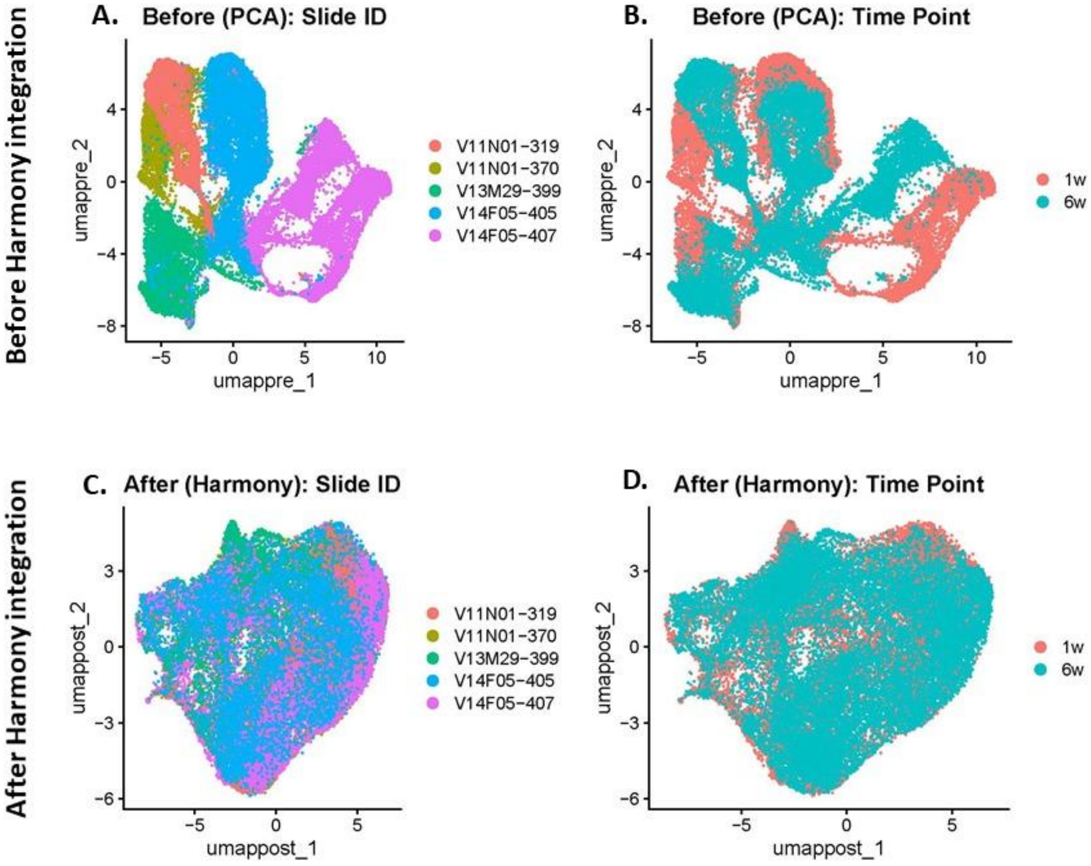

Figure 2. Harmony UMAP plots. In A., most of spots for each slide IDs have distinct clustering. After Harmony correction C., the spots are no longer only clustered by slide IDs, instead the spots from different slides are spread across acquiring a similar over all structure. Before Harmony correction, in B. time point specific spots cluster somewhat to reveal two different structures for 1-week and 6-week data. After Harmony correction in D., all spots are spread across a similar structure but still preserve the clustering by time points as 6-week spots overlay on 1-week.

- No Gene-Level Correction**:** Harmony does not modify gene expression values directly; it changes the positions of cells in a manifold where genes contribute jointly. Hence, no "corrected expression matrix" exists to use for downstream differential or co-expression analyses.

- Goal of Structural Alignment, Not Quantitative Correction**:** Harmony's objective is to align the geometry of the data, ensuring that clusters reflect biological states instead of batch origins. It is designed for visualization, clustering, and trajectory analysis — not for reconstructing corrected gene-level expression.

3.4. Limma

The limma (Linear Models for Microarray Data) framework, originally designed for microarray analysis and later adapted for RNA-seq, provides a statistically rigorous way to model and remove these unwanted technical sources of variation (6). The function removeBatchEffect() from the limma package performs linear modeling-based correction at the gene level, making it interpretable and compatible with downstream differential expression and co-expression analyses with caution/treatment of the output. Limma applies a linear modeling approach to remove unwanted sources of variation while preserving biological signal. Unlike Harmony or Seurat integration, which modify latent embeddings, limma directly operates on normalized gene expression matrices. The key is to use a linear model to partition the expression of each gene into components attributable to biological factors of interest (e.g., time point, condition), technical factors (batch effects) and random noise.

For each gene g, expression is modeled as: $Y\_g = X\beta\_g + \varepsilon\_g$, where Y_g are expression values across all samples or spots, X is the design matrix specifying experimental covariates (biological and batch factors), β_g represents coefficients describing each covariate's contribution, and ε_g is the residual noise. The removeBatchEffect() function projects out variation attributable to specified batch covariates while preserving the biological variation encoded in the design matrix.

3.4.1 Work Flow

A. Normalize and log-transform
   - Expression values are first normalized (e.g., using Seurat's `NormalizeData` or edgeR's `cpm`), then log-transformed.

B. Create metadata and Design matrix
   - Define metadata variables representing both biological and technical covariates

C. Apply batch correction
   - Use `removeBatchEffect`, this function removes unwanted technical variation while preserving the biological signal defined in `design`.

D. Create a corrected Seurat object for visualizations and clustering
   - The corrected matrix can be loaded into Seurat for visualization, clustering, and differential analysis.

In Figure 3, pre-limma UMAP plots show clear clustering in the space for operator and slide ID and time point. Post-limma correction still shows reduced distinct clustering for operator, and the batch effects seem to have mitigated within the samples by the same operator. There is reduction in distinct clustering by slide ID, but still visible clustering due to time points, which means instead of slide IDs, the data are clustering based on similarity of expression between the spots.

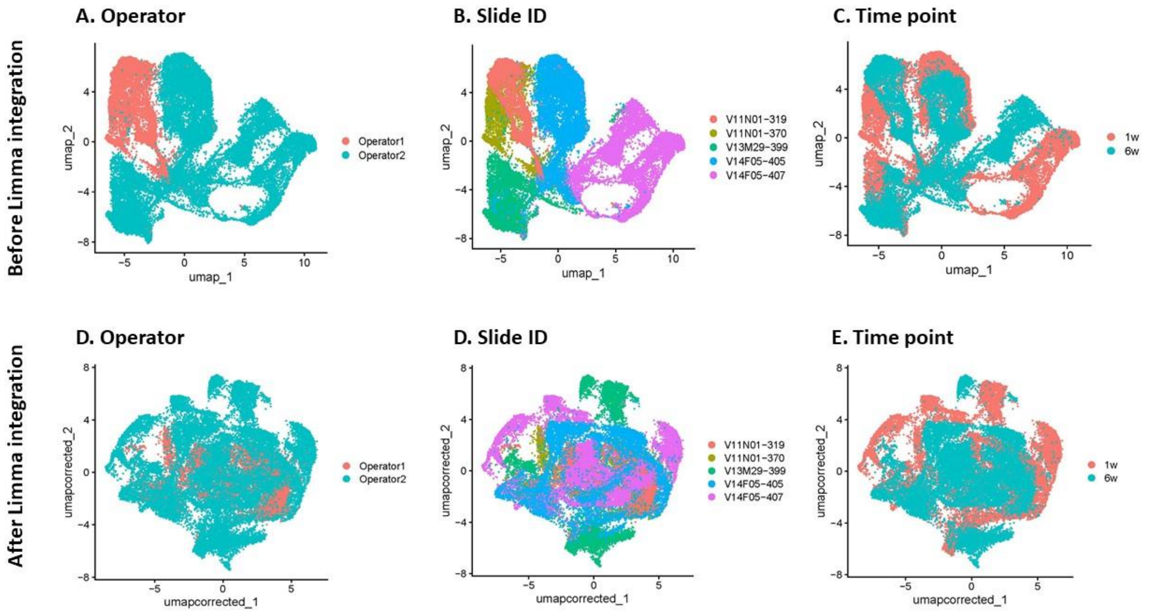

Figure 3. Limma UMAP plots. A, B, C. Pre-Limma UMAPs: The TSNE clusters show clear batch effects for Slide ID and Operator. D, E, F. Post-Limma UMAPs: overall structures improve, but operator clustering is still evident with Operator 2 overlaid non-uniformly on Operator 1 collected data. Distinct clustering due to slide IDs and time point is reduced, but still identifiable.

3.4.2 Limma output is suitable for downstream differential expression analysis, but requires further considerations

Limma assumes linear additive effects of batch and biological covariates and may not fully capture non-linear batch structures. It is also sensitive to unbalanced designs (if certain batches contain only specific conditions) and it does not inherently address latent structure or nonlinear batch effects seen in high-dimensional spaces—where methods like Harmony may perform better. Although the output from Limma is directly applicable for downstream analysis like differential expression analysis, the corrected data assume a linear nature and require additional testing, processing and transformation prior to use in differential expression analysis for accurate results.

3.5. Loupe Browser

Loupe Browser (10x Genomics) is a convenient and user-friendly platform to conduct differential expression analysis on aggregated Visium (10x Genomics) spatial transcriptomics data (7). A detailed description of Loupe Browser methods is provided in the main manuscript under “2.3.1 Differential expression analysis of sequenced genes”.

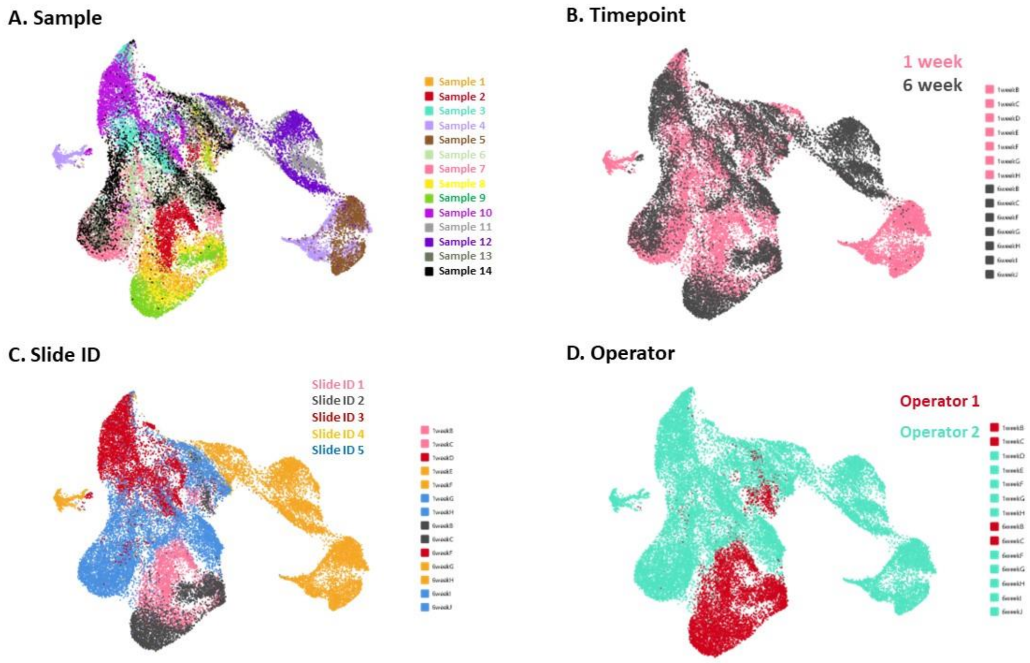

Figure 4. Loupe Browser (10x Genomics) UMAPs. A, C. Sample and Slide ID clusters are identifiable and spread across the data structure. B. Timepoint clustering is relatively reduced, and more widespread. D. Operator batch effects are evident with some overlay of Operator 2 on Operator 1 in the two-dimensional space.

We inspected UMAPs of the raw data in Loupe Browser to check for batch effects. Although an operator batch effect is evident in Figure 4, the data is overlaid, and other variables (Slide ID/time point) had relatively less observable batch effects on the data (clusters exist as well as expand across the structure). Furthermore, we were able to select the regions of interest for differential expression analysis and inspect the number and location of the selected spots on the UMAP. We proceeded with differential expression analysis using Loupe Browser for this study after certain considerations: (1) the number of spots selected for differential expression (~540 spots) was a relatively low (as opposed to all available spots mapping the tissue section ~32,900 spots) and included an even smaller number of spots (78 spots) generated from the "Operator 1"-affected samples, (2) the use of additional correction and normalization performed by the software prior to differential expression analysis, and (3) the ease and efficiency of the Loupe Browser software and data aggregation pipeline.

References

1. Whitsitt Q, Saxena A, Patel B, Evans BM, Hunt B, Purcell EK. Spatial transcriptomics at the brain-electrode interface in rat motor cortex and the relationship to recording quality. J Neural Eng. 2024 Aug 1;21(4):046033. doi:10.1088/1741-2552/ad5936

2. Hao Y, Hao S, Andersen-Nissen E, Mauck WM, Zheng S, Butler A, et al. Integrated analysis of multimodal single-cell data. Cell. 2021 Jun;184(13):3573-3587.e29. doi:10.1016/j.cell.2021.04.048

3. Satija Lab. GitHub. 2021. Differential expression testing post-integration #4000 | GitHub.

4. Hoffman P, Satija Lab and Collaborators. Seurat 4.3.0 [Internet]. 2023 [cited 2026 Apr 5]. Introduction to scRNA-seq Integration | Seurat 4.3.0. Available from: https://satijalab.org/seurat/archive/v4.3/integration_introduction

5. Korsunsky I, Millard N, Fan J, Slowikowski K, Zhang F, Wei K, et al. Fast, sensitive and accurate integration of single-cell data with Harmony. Nat Methods. 2019 Dec 18;16(12):1289–96. doi:10.1038/s41592-019-0619-0

6. Ritchie ME, Phipson B, Wu D, Hu Y, Law CW, Shi W, et al. limma powers differential expression analyses for RNA-sequencing and microarray studies. Nucleic Acids Res. 2015 Apr 20;43(7):e47–e47. doi:10.1093/nar/gkv007

7. LoupeBrowser | 10xGenomics [Internet]. [cited 2025 Apr 12]. Available from: https://www.10xgenomics.com/support/software/loupe-browser/latest
